# Phase Effects in Zirconia Catalysed Glucose Conversion to 5‐(Hydroxymethyl)furfural

**DOI:** 10.1002/cssc.202401494

**Published:** 2024-11-21

**Authors:** Yang Liu, Luke Forster, Aristarchos Mavridis, Andrea Merenda, Mohamed Ahmed, Carmine D'Agostino, Muxina Konarova, Aaron Seeber, Enrico Della Gaspera, Adam F. Lee, Karen Wilson

**Affiliations:** ^1^ School of Science RMIT University Melbourne VIC 3000 Australia; ^2^ Department of Chemical Engineering The University of Manchester Manchester M13 9PL UK; ^3^ Australian Research Council Research Hub for Nutrients in a Circular Economy Centre for Technology in Water and Wastewater School of Civil and Environmental Engineering University of Technology Sydney, NSW 2007 Australia; ^4^ Faculty of Engineering and Information Technology University of Technology Sydney Ultimo NSW 2007 Australia; ^5^ School of Chemical Engineering The University of Queensland Brisbane, QLD 4072 Australia; ^6^ Dipartimento di Ingegneria Civile, Chimica, Ambientale e dei Materiali (DICAM) Alma Mater Studiorum - Università di Bologna 40131 Bologna Italy; ^7^ CSIRO Manufacturing Research Way Clayton Melbourne VIC 3168 Australia; ^8^ Centre for Catalysis and Clean Energy Griffith University Gold Coast QLD 4222 Australia

**Keywords:** Catalytic biorefining, Zirconia, Solid acid, Glucose, Fructose, 5-(hydroxymethyl)furfural, Flow chemistry

## Abstract

5‐(hydroxymethyl)furfural (HMF) is a key biomass derived platform chemical used to produce fuel precursors or additives and value‐added chemicals, synthesised by the cascade isomerisation of glucose and subsequent dehydration of reactively formed fructose to HMF over Lewis and Bronsted acid catalysts, respectively. Zirconia is a promising catalyst for such reactions; however, the impact of acid properties of different zirconia phases is poorly understood. In this work, we unravel the role of the zirconia crystalline phase in glucose isomerisation and fructose dehydration to HMF. The Lewis acidic monoclinic phase of zirconia is revealed to preferentially facilitate glucose isomerisation, while the nanoparticulate tetragonal phase possesses Brønsted acid sites which favour fructose dehydration. Synergy between both zirconia phases facilitates cascade HMF production, with both catalysts investigated as physical mixtures in batch and flow reactor configurations. Using a physical mixture of only 15 wt % *m‐*ZrO_2_ with 85 wt % *t‐*ZrO_2_ in either batch or packed bed reactor configuration is sufficient to reach equilibrium conversion of glucose for subsequent dehydration by the *t‐*ZrO_2_ component. Under continuous flow, a six‐fold increase in HMF production was obtained when operating with a physical mixture of *m‐* and *t‐*ZrO_2_ compared to that from a single bed of *t‐*ZrO_2._

## Graphical abstract

Phase matters: The tuneable surface properties of nanoparticulate zirconia polymorphs is exploited for catalytic cascade reactions of biomass derived molecules. Monoclinic and tetragonal zirconia possessing predominantly Lewis and Brønsted acid sites respectively drive the tandem isomerisation of glucose to fructose and subsequent dehydration to 5‐HMF under continuous flow.

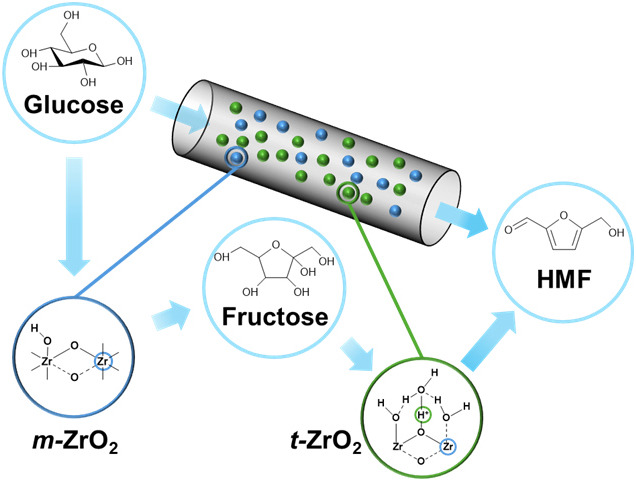

## Introduction

The impact of anthropogenic carbon dioxide on climate change, and the pursuit of sustainable resources to meet the demand of a rapidly growing global population, necessitate urgent decarbonisation of the chemical industry, including a transition to renewable carbon resources. Biorefineries are the most promising solution to sustainable chemical manufacturing, wherein waste biomass or plastics are transformed to energy vectors (e. g. liquid hydrocarbons and H_2_) and chemical building blocks.^[1]^ The most abundant biomass source is lignocellulose from forestry and agricultural waste, although offshore algae farms which produce bio‐oils also experience a periodic resurgence of academic and commercial interest. Despite its abundance, the valorisation of lignocellulosic waste remains challenging, requiring fractionation into its constituent lignin and holocellulose components and their subsequent depolymerisation into phenolic derivatives and pentose/hexose monomers, respectively.^[2]^ Recent developments in ‘lignin‐first’ biomass processing, such as reductive catalytic fractionation, permit the simultaneous separation and depolymerisation of lignin from cellulose pulp and hence are technologically superior to steam explosion (which produces recalcitrant forms of lignin and inhibitory carbohydrate by‐products).

**Scheme 1:**
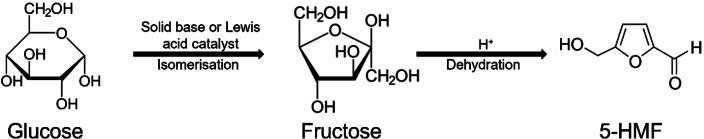
Cascade isomerisation and dehydration of glucose to 5‐HMF.

5‐(Hydroxymethyl)furfural (HMF) is one of the most valuable chemical products obtainable from holocellulose, and designated as one as a key platform chemical by the US Department of Energy.^[3]^ HMF is a precursor to eco‐friendly solvents^[4]^ and polymers,^[5]^ and linear alkanes to grow hydrocarbon chains to sustainable diesel and kerosene fuels.^[6]^ The catalytic synthesis of HMF from fructose can be effectively achieved in biphasic solvents, such as water/DMSO wherein 68 % HMF (and complete fructose conversion) is reported at 170 °C in 4 h,^[7]^ or ionic liquids including 1‐ethyl‐3‐methylimidazolium bromide wherein 75 % HMF was obtained for 10 wt % fructose in 2 h at 100 °C using a homogeneous SnCl_4_ catalyst.^[8]^ However, fructose is only found as fructans (such as inulin) in <15 % of flowering plants and features in human and animal diets, and hence is not prevalent in waste biomass. In contrast, glucose is abundant from cellulose in grass and woody biomass waste, and the preferred low cost feedstock for HMF through a cascade isomerisation to fructose and subsequent dehydration (Scheme 1).^[9]^ The combination of Lewis acidic CrCl_3_ and Brønsted acidic HCl achieved a 59 % HMF yield in 3 h at 140 °C using a biphasic (water/THF) solvent with NaCl promoter.^[10]^ HCl catalysed glucose conversion in a water/γ‐valerolactone solvent (with a NaCl promoter) delivered a 62 % HMF yield in 1 h at 140 °C.^[11]^ Bifunctional organocatalysts such as sulfanilic acid are also reported for this cascade, albeit requiring a multiphasic MIBK/DMSO/water solvent and 160 °C to attain 44 % HMF in 0.5 h.^[12]^ Note a pure Brønsted acid (HCl) catalysed direct route to HMF through glucose dehydration in methyl isobutyl ketone (MIBK):water (96 : 4) solvent was recently reported;^[13]^ this produced 74 % HMF at 125 °C in 3 h, facilitated by acyclic isomerisation and dehydration favoured by ketone‐based organic solvents, and avoiding the need for less active and stable (and more toxic) Lewis acids for the initial isomerisation of glucose. However, the use of homogeneous catalysts is problematic due to the challenges of product separation (catalyst recycling),^[14]^ safety, requirement for corrosion‐resistant reactors, and scale‐up.^[15]^ Early efforts to introduce solid Lewis acidity through Sn‐Beta zeolite showed promise with 11 wt % HMF yield in 2 h at 140 °C from a 10 wt % glucose aqueous solution, albeit with concentrated HCl to catalyse fructose dehydration. Fully heterogeneous catalysts for the two‐step conversion of glucose to HMF through isomerisation to fructose (over base/Lewis acid sites) and subsequent fructose dehydration to HMF (over Brønsted acid sites) are widely reported,[^16^, ^17^, ^18^] and permit continuous operation, aligning with sustainable chemical manufacturing practices.^[19]^

Glucose isomerisation to fructose, a process integral to the production of high‐fructose corn syrups, representing the most extensive commercial biocatalytic operation,^[20]^ may occur via different acid‐catalysed pathways. The Brønsted base pathway follows a Lobry de Bruyn‐van Ekenstein rearrangement, in which glucose reacts with a base to form the 1,2‐enediol, with a subsequent proton transfer to yield fructose.^[21]^ However, this pathway is prone to by‐product formation, including oligomers via condensation and saccharinic acid (by intramolecular redox processes), and hence low fructose selectivity.^[22]^ The Lewis acid catalysed pathway involves an intramolecular hydride shift, demonstrated by Román‐Leshkov and co‐workers over a Sn‐β zeolite,^[23]^ wherein the glucose ring‐opening occurs through hydrogen transfer from C‐2–C‐1 and O‐2–O‐1 functions.^[24]^ Although isomerisation in water helps solubilise the sugars and offers a green solvent, thermodynamics cap the maximum fructose yield ~57 % at 100 °C in pure water due to the reverse reaction (fructose hydrolysis).^[25]^ Fructose dehydration to HMF is catalysed by Brønsted acids, and involves the elimination of three water molecules. Various solvent systems have been studied, including biphasic water‐organic,^[26]^ eutectics,^[27]^ and ionic liquids.^[28]^ As HMF is thermally unstable >100 °C,^[29]^ its separation is challenging from higher boiling point organic solvents, with distillation often resulting in undesired soluble oligomers or humins.

Zirconia (ZrO_2_) has been widely used as a catalyst or catalyst support for biomass conversion due to its amphoteric surface chemistry, phase dependent surface oxygen vacancies^[30]^ and hydrothermal stability,^[31]^ which render zirconias promising catalytic material for the aqueous phase conversion of glucose to HMF.[^32^, ^33^, ^34^] ZrO_2_ calcined at 400 °C is reported to yield 35 % HMF from 3 wt % of glucose in 8 h at 170 °C, but using a biphasic DMSO/H_2_O solution wherein DMSO promoted fructose dehydration.^[35]^ Doping of ZrO_2_ with oxyanions such as SO_4_^2−^ can stablise the tetragonal phase, and thereby enhance Brønsted acid strength. For example, SO_4_/ZrO_2_ (SZ) with 1.5 wt % SO_4_ (equivalent to 0.3 monolayers) offers dual Lewis and Brønsted acidity, resulting in 78 % and 5 % selectivity to fructose and HMF, respectively in water.^[17]^ The catalytic activity of zirconia is dependent on surface area and active site accessibility,^[36]^ with conformal monolayers of SZ grown on a SBA‐15 mesoporous silica framework enhancing aqueous HMF productivity three‐fold versus SZ nanoparticles.^[37]^ A similar ZrO_2_/MCM‐41 mesoporous silica catalyst exhibited 82 % glucose conversion and 23 wt % HMF yield after 2.5 h at 175 °C, but in a MIBK/H_2_O solvent system.^[38]^ Chongwen and coworkers also achieved 79 % glucose conversion and 35 % HMF yield (albeit for a very low glucose:catalyst mass ratio of 4) using zirconium doped KIT‐6 (~5 mol % Zr) in MIBK/H_2_O in 3 h at 170 °C.^[39]^ Ceria‐doped SZ/SBA15 (7 wt % Zr and 1.6 mmol g^−1^ S) afforded 67 % HMF yield at 71 % selectivity in 6 h at 120 °C, employing a 90 vol % isopropanol/DMSO solvent,^[40]^ however isopropanol is not readily obtained from biomass and hence its ‘green’ credentials are debatable.^[41]^ In the aqueous phase, a 2 wt % Pd promoted ZrO_2_ reportedly achieved 55 % glucose conversion and 74 % HMF selectivity in 3 h at 160 °C (30 bar autogeneous pressure), with a TOF of 0.6 h^−1^.^[42]^ The preceding literature describe batchwise reactions, although continuous flow reactions offer superior heat and mass transfer, and can significantly improve space‐time yields.^[43]^ Clayton and coworkers report a 21 % HMF yield from a high concentration (23 % wt %) of glucose reacted in MIBK/H_2_O passed over commercial monoclinic (*m−)*ZrO_2_ in a fixed‐bed flow reactor at 180 °C (2 min residence time);^[44]^ unfortunately, no kinetic parameters or deactivation profiles were detailed. Continuous flow production of HMF from glucose is reported over forms of zirconia (PO_4_/ZrO_2_) catalysts in water,^[45]^ or alternative inorganic/resin catalysts in mono‐^[46]^ or biphasic^[47]^ organic solvent systems, but to our knowledge is not reported over ZrO_2_ catalysts in the aqueous phase.

Here we explore the influence of zirconia phase and reactor configuration on the cascade conversion of glucose to HMF, using well‐defined monoclinic and tetragonal zirconia nanocrystals. The former *m‐*ZrO_2_ crystallised as larger particles with a lower surface area and exposed Lewis acid sites active for the aqueous phase isomerisation of glucose to fructose at 100 °C, while the latter *m‐*ZrO_2_ exposed Brønsted acid sites active for fructose dehydration to HMF under the same conditions. Combinations of both phases promoted the overall cascade, with physical mixture in a packed bed, continuous flow reactor delivering the optimal HMF yield, with negligible catalyst deactivation over 6 h on‐stream. This work highlights both the importance of phase engineering in solid acid catalysis, and flow chemistry for intensified processing.

## Experimental Section

### Materials and Synthesis

Zirconium oxynitrate hydrate (ZrO(NO_3_)_2_⋅xH_2_O, technical grade, 99 %), urea (ACS reagent, 99.0–100.5 %), ammonia hydroxide solution (≥25 % NH_3_ in H_2_O), glucose (≥99.5 %), fructose (≥99 %), 5‐HMF (analytical standard), DMSO (≥99.9 %) were supplied by Sigma Aldrich. Zirconium hydroxide (MEL chemical, XZO1247) was supplied by MEL Chemicals (now Luxfer). H_2_O (milliQ, Millipore) were used as the reaction solvent. Monoclinic and tetragonal zirconia were prepared with zirconyl oxynitrate using a hydrothermal and reflux method respectively, according to the following protocols. For monoclinic ZrO_2_ (*m*‐ZrO_2_), 13.9 g zirconyl oxynitrate and 36 g urea were dissolved in 100 mL milli‐Q water, then the solution was transferred into 200 mL Teflon‐lined stainless‐steel autoclave (RobotDigg, Shanghai) and hydrothermally treated at 180 °C for 20 h under static conditions. The slurry was filtered and washed with water and acetone until pH 7 before drying in the oven at 80 °C overnight. The product was calcined in a muffle furnace under static air at 800 °C for 4 h with a ramping rate of 5 °C min^−1^. Tetragonal ZrO_2_ (*t*‐ZrO_2_) was prepared by adding 6.9 g zirconium oxynitrate and 5.2 g P123 (P123:Zr molar ratio ~0.03) in 200 mL milli‐Q water. The pH was adjusted to 11 using NH_3_⋅H_2_O after the mixture was fully dissolved. The slurry was refluxed in a 500 mL round bottom flask with stirring for 40 h at 88 °C. The product was filtered, washed with water and acetone to remove the template. After drying in the oven at 110 °C for 24 h, the product was calcined under static air in a muffle furnace at 700 °C for 4 h with a ramping rate of 5 °C min^−1^. Mixed phase zirconia was synthesised by calcining zirconium hydroxide in the muffle furnace under static air at 400 °C for 4 h with a ramping rate of 5 °C min^−1^. The as‐prepared catalysts were stored in air and used without any pretreatment.

### Catalyst Characterization

Powder X‐ray diffraction (XRD) patterns were collected in the range 2θ=10–80° with a step size of 0.02° on a Bruker D8 Advance A25 X‐ray diffractometer equipped with a Lynx Eye XE−T detector, using Cu K_α_ radiation (40 kV, 40 mA). Crystalline phases were identified using the ICDD‐JCPDS powder diffraction database. Phase qualification and crystallite size values were determined via the Rietveld refinement using the Bruker TOPASTM V6 program. Background was described using a combination of Chebyshev polynomial linear interpolation function and 1/x function. Material morphologies and lattice spacing were examined by transmission electron microscopy (TEM) using a JEOL JEM‐2100F TEM operated at 200 kV, equipped with a Gatan OneView 4 k camera. Surface area and pore size analysis was performed by N_2_ physisorption on a Novatouch LH4 porosimeter at 77 K, after degassing ~90 mg of catalyst which was weighed accurately in the porosimetry cell and outgassed under vacuum for 3 h at 120 °C. Full isotherms were acquired over the pressure range P/P_0_=0.02–1, with data processed through Quantachrome Touch Win (Version 1.22) software. Surface areas were calculated using the Brunauer‐Emmett‐Teller (BET) method over the range P/P_0_=0.05–0.24, where a linear relationship was maintained, while pore size distributions were calculated by using Barett‐Joyner‐Halenda (BJH) method applied to the desorption branch of the isotherm. X‐ray photoelectron spectroscopy (XPS) measurements were performed using Thermo K‐alpha photoelectron spectrometer equipped with a hemispherical analyser and Al Kα (hν=1486.7 eV) X‐ray source. Survey scans were collected under vacuum over a range of 0–1350 eV with a step size of 1 eV and a pass energy of 200 eV. High resolutions scans were acquired at 50 eV pass energy with a step size of 0.1 eV. Data was processed using CasaXPS software (version 2.3.24, Casa Software Ltd., Teignmouth, UK), with binding energies (BE) calibrated against C 1s signal (BE=284.8 eV) and high‐resolution O 1s and Zr 3d XP spectra fitted using a Lorentzian line shape. Shirley backgrounds were subtracted from all high‐resolution spectra. Surface composition was quantified by applying specific response sensitivity factors (RSF) of C 1s, O 1s and Zr 3d, with values of 1.0, 2.93 and 7.04, respectively. Acidity measurements were performed by diffuse reflectance infrared fourier transform (DRIFT) spectra of adsorbed pyridine, recorded at ambient conditions on a Perkin Elmer FTIR instrument equipped with a Perkin Elmer Diffuse Reflectance Sampling Accessory. Spectra was scanned from 400–4000 cm^−1^ with a resolution of 4 cm^−1^ and 32 accumulation scans. 50 mg samples were impregnated in 100 μL pyridine without any pretreatment and stored in vacuum oven at 30 °C overnight to remove any excess of pyridine prior to samples loaded for measurement. Acid/base site loadings were measured via NH_3_/CO_2_ chemisorption and temperature‐programmed desorption (TPD) on a Micromeritics ASAP2029 chemisorption instrument. For a typical TPD experiment, 100 mg of catalysts was pretreated at 500 °C for 30 min and then cooled to 100 °C under flowing He (50 mL min^−1^). NH_3_/CO_2_ adsorption was performed at 100 °C for 20 min with a gas flow rate of 30 mL min^−1^, followed by a He flush to remove any excess NH_3_/CO_2_. The furnace temperature was subsequently increased to 700 °C with a ramp rate of 10 °C min^−1^ with desorbed NH_3_/CO_2_ monitored by a thermal conductivity detector (TCD). The acquired data was analysed against instrument calibration to quantify acidity/basicity. NMR relaxation was performed on a Spinsolve™ instrument, with a static magnetic field of around 1 T, corresponding to a proton Larmor frequency of roughly 43 MHz. Samples were prepared by soaking the catalysts in the liquid (n‐Octane, Pyridine, THF and water) overnight to fully saturate the surface. Prior to the measurements, the samples were removed from the liquid, and carefully dried using filter papers to remove any liquid layers from the external surfaces between the solid grains. A standard T1‐T2 NMR pulse sequence (Figure S11) was employed on the samples to determine spin‐lattice and spin‐spin relaxation times. The sequence was repeated for 32 different delay times, which were separated by log‐spaced intervals. An echo time of 120 μs was chosen, sufficiently short to minimise internal magnetic field gradient effects. The raw relaxation data obtained from these measurements have been inverted using an algorithm utilising Tikhonov regularisation, with the regularization parameter determined through generalized cross‐validation.

Analysis of catalysts post‐reaction was performed after filtering and drying in the oven at 80 °C overnight. The carbon species on the surface of spent catalysts were analysed via temperature‐programmed oxidation (TPO) carried out on a Netzsch STA 449 F3 Jupiter® thermal analyser connected to a Netzsch 403 Aëolos® Quadro quadrupole mass spectrometer, using a compressed air flow of 10 mL min^−1^ while heating from 35–800 °C with a ramp rate of 10 °C min^−1^. The carbon species decomposition was examined by following the 44 amu mass spectrometry (MS) signal for CO_2_, with peak deconvolution performed using a Gauss function.

### Catalytic Tests

Batch: Kinetic studies of glucose and fructose conversion were conducted in a Radleys Starfish carousel reactor under stirring with 450 rpm at 100 °C for 6 h. In a typical procedure, 0.1 g glucose or fructose and 50 μL DMSO (as an internal standard for HPLC analysis) were dissolved in 20 mL Milli‐Q water (equivalent to 28 mM), once the temperature of reactant reached 100 °C, 0.2 g catalyst was added to the reaction mixture. Flow: Continuous flow glucose reaction was conducted at 100 °C using a Uniqsis FlowSyn reactor equipped with two high‐pressure pumps. 100 mg (single bed) or 100 mg of each catalyst (physical mixture and dual‐bed) was diluted with silicon carbide (AussieSapphire, mesh size=80) and packed within a 10 mm id×100 mm OMNIFIT® glass column (Cross‐sectional area=0.785 cm^2^) to give a total bed length between 4.5 and 5.5 cm and volume between 3.5 and 4.3 cm^3^. A liquid reactant stream of Milli‐Q water (200 mL), glucose (1 g), and DMSO (0.5 mL), equivalent to 28 mM glucose solution, was delivered by the integrated HPLC pumps at flow rate of 0.4 (τ of 10 min) or 0.08 (τ of 50 min) mL.min^−1^ as calculated using the equation Flow rate (Q)=catalyst bed volume (V)/residence time (RT). Aliquots were collected periodically and filtered with 0.22 μm nylon syringe filter prior to analysis on a Perkin Elmer Flexar HPLC equipped with Waters refractive index detector and UV diode array detectors, and an Agilent Hi‐Plex H column. A 5 mM sulphuric acid solution was used as the mobile phase, with a flow rate of 0.6 mL min^−1^ and 65 °C column temperature. Reactant conversion and product yields were calculated using response factors determined by multi‐point calibration of HPLC standards. Conversion, yield and selectivity were calculated according to equations [Equations (1–3)], while errors were experimentally determined as ±5 % based on the standard deviation of triplicate measurements.

(1) $$Reactant\;conversion\;\left(\%\right)=\;\frac{C_{reactant,t=0}-C_{reactant}}{C_{reactant,t=0}}\times 100$$

(2) $$Yield\;of\;product\;n\;\left(\%\right)=\frac{C_{n}}{C_{reactant,\;t=0}}\times 100$$

(3) $$Selectivity\;to\;product\;n\;\left(\%\right)=\frac{C_{n}}{C_{reactant,\;t=0}-C_{reactant}}\times 100$$

where C_n_ is the molar concentration of species n.

Initial reaction rates in batch reactions were examined using data points of the first hour where a good linear relationship was maintained and the conversion is <20 %.^[48]^ In some instances, activities are also reported normalised per surface area of catalysts.

Turnover frequencies (TOFs) were calculated according to the following equation [Equation 4]:

(4) $$TOF=\;\frac{Initial\;rate\;of\;glucose\;consumption\;(mols.h^{-1})}{Number\;of\;surface\;acid\;sites\;\left(mols\right)}$$

## Results and Discussion

### Catalyst Characterisation

**Figure 1:**
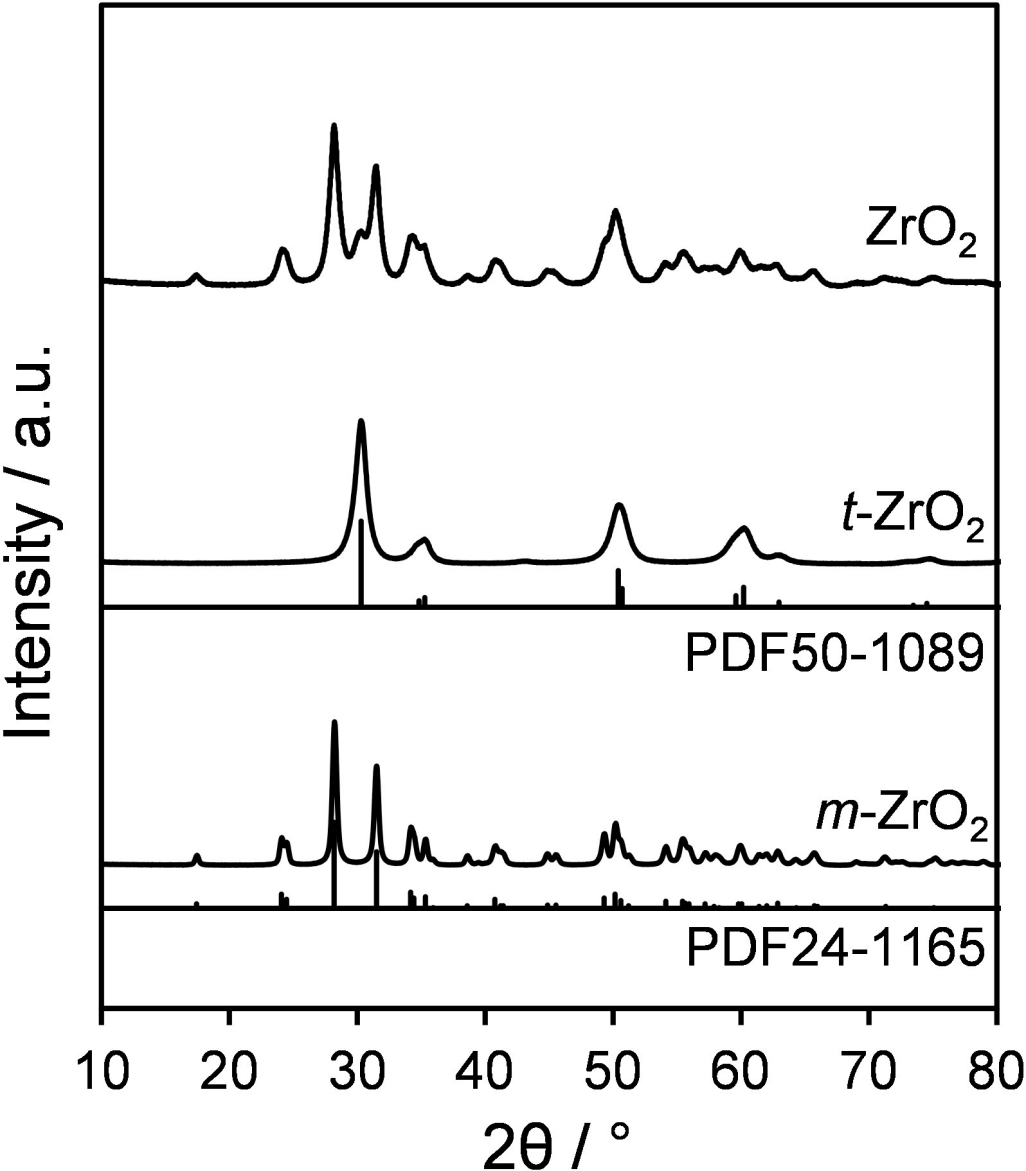
Powder XRD of tetragonal (*t*−) and monoclinic (*m*−) ZrO_2_ nanoparticles and mixed phase ZrO_2_ prepared by calcination of Zr(OH)_4_ at 400 °C.

Successful synthesis of phase pure monoclinic (*m−)* and tetragonal (*t−)* zirconia nanoparticles was first confirmed by powder XRD and Rietveld refinement analysis. Figure 1 shows typical XRD patterns of as‐prepared, nanoparticulate *t*‐ and *m*‐ZrO_2_, and mixed phase ZrO_2_ (ZrO_2_, prepared by calcination of Zr(OH)_4_ at 400 °C). Both *t*‐ and *m*‐ZrO_2_ showed reflections matching reference data (ICDD) with phase purities >99 % determined by Rietveld analysis (Table S1), whereas ZrO_2_ comprised 86 wt % and 14 wt % *m‐* and *t‐* phases, respectively. Scherrer analysis determined the volume‐averaged crystallite sizes of pure *t*‐ZrO_2_ and *m*‐ZrO_2_ as 6.7 nm and 17.9 nm respectively, while the corresponding crystallite size of *t‐* and *m‐* components within the mixed phase ZrO_2_ were 8.0 and 8.6 nm, respectively. The phase stability of ZrO_2_ is particle size dependent, with bulk lattice energies favouring *m‐*ZrO_2_ for larger crystallites, whereas surface energies favour *t‐*ZrO_2_ for crystallites <9 nm, consistent with our observations.^[49]^

**Figure 2:**
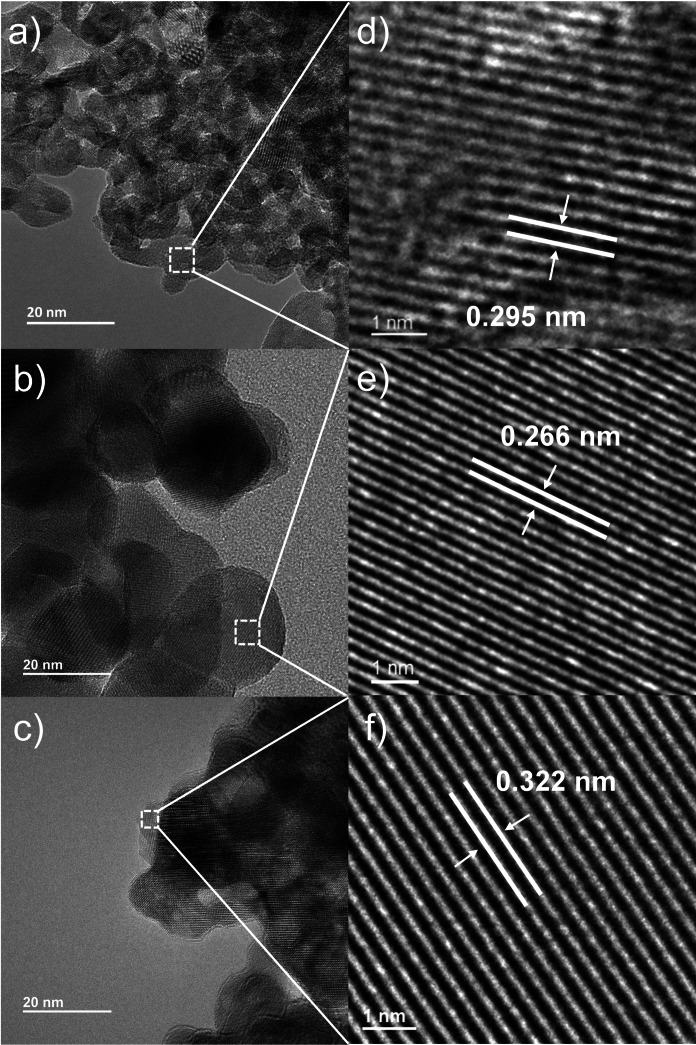
HRTEM images of (a) *t‐*ZrO_2_, (b) *m‐*ZrO_2_ and (c) ZrO_2_ with magnified regions showing (d) (101) plane of *t‐*ZrO_2_, e) (002) plane of *m‐*ZrO_2_, and f) the (11$\bar{1}$ ) plane of *m‐*ZrO_2_ in the mixed phase material.

High‐resolution transmission electron microscopy (HRTEM) was subsequently used to analyse the morphology and crystallinity of the prepared materials. All *t‐*ZrO_2_ (Figure 2a and d) and *m*‐ZrO_2_ (Figure 2b and e) particles were rather isotropic in shape, with sizes in agreement with the average from XRD, suggesting that the particles observed by TEM are mainly single crystals. High‐resolution images of mixed phase ZrO_2_ (Figure 2c and f) revealed lattice fringes with a d‐spacing of 0.295 nm, attributed to the (101) plane of *t‐*ZrO_2_, which was also observed at 2θ=30.2° in Figure 1. The d‐spacings of 0.266 nm (Figure 2d) and 0.322 nm (Figure 2f) correspond to the (002) and (11$\bar{1}$ ) planes of the monoclinic phase, which were also observed at 2θ=34.2° and 28.2° respectively in Figure 1. Tetragonal phase particles were not observed in ZrO_2_, presumably due to their lower wt % within the material. Additional TEM images and particle size distributions (Figure S1) indicate relatively homogeneous materials, with sizes of 8±1.5 nm for *t‐*ZrO_2_ and 30±5 nm for *m*‐ZrO_2_. Indexing of Fast Fourier transform FFT processed images (Figures S1d, h and l) reveals the presence of (111), (011), (11$\bar{1}$ ) and (100) planes of monoclinic zirconia for *m‐*ZrO_2_ and ZrO_2_, and (101) and (021) planes for *t‐*ZrO_2_. Raman spectroscopy confirmed the presence of monoclinic zirconia in *m‐*ZrO_2_ and ZrO_2_ indicated by bands at 176, 189, 305, 331, 346, 382, 475, 501, 536, 559, 615, and 637 cm^−1^ (Figure S2),^[50]^ and of the tetragonal phase in for *t‐*ZrO_2_ indicated by at 146, 274, 312, 459 and 645 cm^−1^.^[51]^ The corresponding band assignments are detailed in Table S2.^[52]^

Nitrogen porosimetry reveals all zirconias exhibited type IV isotherms (Figure S3) with hysteresis loops attributable to the presence of interparticle voids (formed by the sintering of particles due to the calcination).^[17]^ The hysteresis loop of *m*‐ZrO_2_ is shifted to higher p/p_0_ compared to those of *t*‐ZrO_2_ and ZrO_2_, suggesting an increase in the mean mesopore diameter of the former (i. e. larger voids) due to larger particle size and less dense packing of the monoclinic phase (Figure 2b). Specific surface areas were comparable for *t*‐ZrO_2_ and ZrO_2_, and almost seven‐fold greater than that of *m*‐ZrO_2_ (Table 1). Pore size and pore volume calculated by BJH method represent the size of interparticle voids (Table S3).^[17]^

**Table 1:** Physicochemical properties of zirconia catalysts.

Catalyst	O : Zr atomic ratio^[a]^	Specific surface area^[b]^ /m^2^ g^−1^	Base loading^[c]^/mmol.g^−1^	Acid loading^[d]^ /mmol.g^−1^	Weak acid site density^[e]^ /μmol.m^−2^	Medium acid site density^[e]^ /μmol.m^−2^	Strong acid site density^[e]^ /μmol.m^−2^	Surface OH loading^[f]^ /mmol.g^−1^
ZrO_2_	2	127	0.41	0.39	0.73	1.74	0.58	0.88
*t*‐ZrO_2_	2.47	153	0.11	0.43	0.89	1.71	0.17	0.54
*m*‐ZrO_2_	2.22	22	0.10	0.10	1.46	2.46	0.50	0.11

[a]XPS. [b]BET method. [c]CO_2_‐TPD. [d]NH_3_‐TPD. [e]Integrated NH_3_‐TPD areas: weak 100–200 °C; medium 200–400 °C; strong 400–600 °C. [f]TGA mass loss between 150 and 500 °C assuming 2Zr‐OH→H_2_O+Zr‐O−Zr.

**Figure 3:**
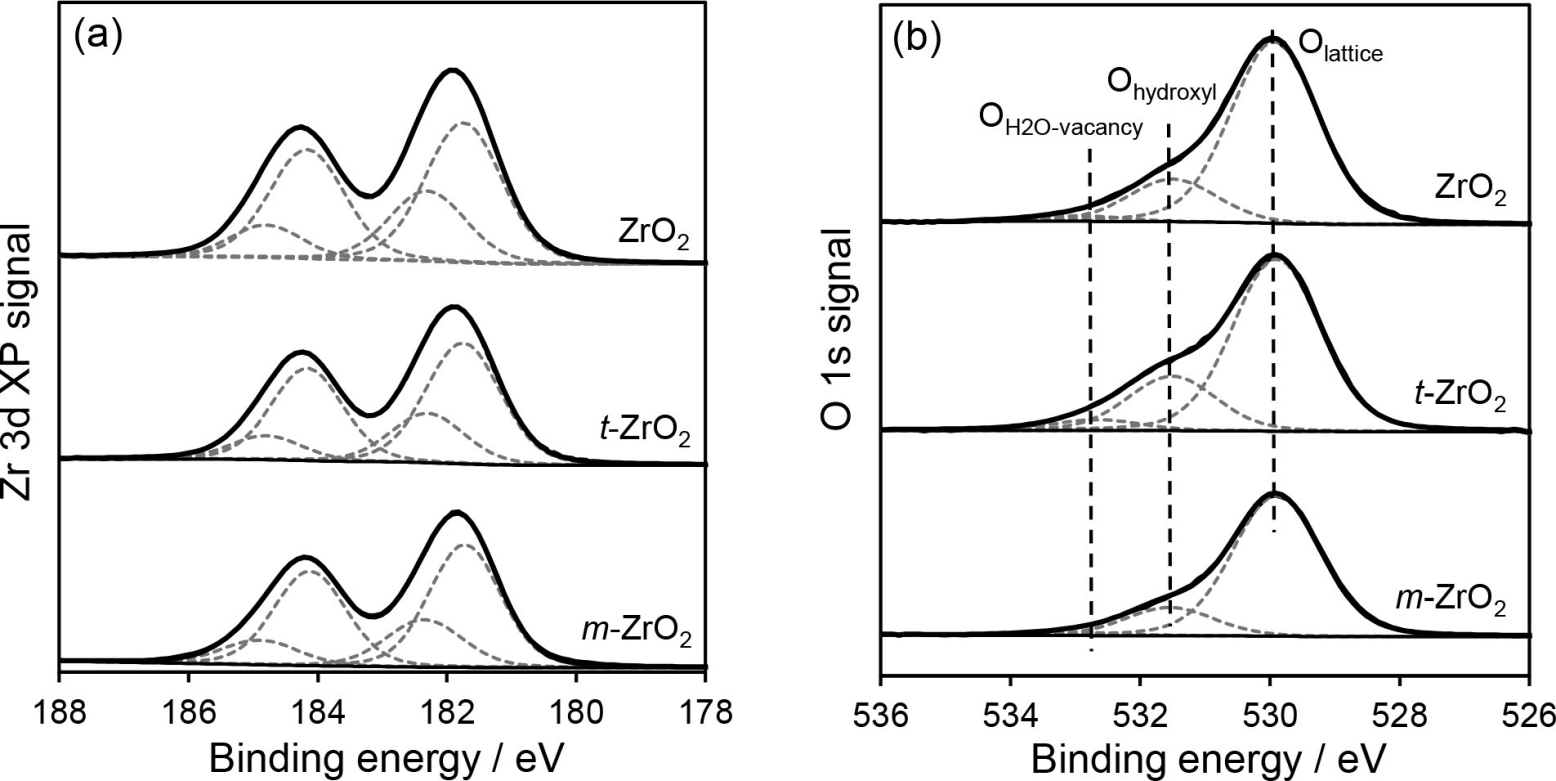
(a) Zr 3d and (b) O 1s XP spectra of ZrO_2_, *t*‐ and *m*‐ZrO_2_ nanoparticles.

XPS analysis revealed identical Zr^4+^ environments in all zirconias (Figure 3a), with Zr 3d_5/2_ and 3d_3/2_ spin‐orbit split components attributable to Zr^4+^ in ZrO_2_ (with binding energies of 181.8 eV and 184.2 eV respectively), and a higher binding energy doublet at 182.3 and 184.8 eV attributed to Zr^4+^ in Zr‐OH.^[53]^ The molar ratio of Zr(OH)_x_:ZrO_2_ was similar in all cases, being 0.36 (ZrO_2_), 0.34 (*m*‐ZrO_2_), and 0.33 (*t*‐ZrO_2_). Corresponding O 1s spectra evidenced three surface species (Figure 3b), characteristic of lattice oxygen (529.9 eV), hydroxyls (531.5 eV), and a weak peak at 532.7 eV attributed to irreversibly adsorbed water at anion vacancies.^[54]^ The O : Zr atomic ratio of both pure phase samples exceeded the expected stoichiometric ratio (Table 1), evidencing surface hydroxyl formation at low coordination sites, whereas that of ZrO_2_ equalled two, consistent its lower surface Zr‐OH coverage from DRIFTS (Figure S4)^[55]^ and the O_hydroxyl_:O_lattice_ ratio of *t‐*ZrO_2_ (0.32)>*m‐*ZrO_2_ (0.24)≥ZrO_2_ (0.23).

Nanoparticulate zirconias may expose various surface sites, including coordinatively unsaturated, Lewis acid‐base (Zr^4+^‐O^2−^) pairs and Brønsted acidic hydroxyls, whose concentrations will depend on the phase and exposed facet.[^56^, ^57^, ^58^] Terminal OH groups observed at 3780–3760 cm^−1^ are typically bound to a single cation, whereas multi‐coordinated OH groups are observed <3740 cm^−1^ and formed on low‐index crystal faces due to steric constraints.^[59]^ Previous studies have shown that low surface area *m‐*ZrO_2_ (6 m^2^ g^−1^) exhibited terminal (3770 cm^−1^) and tri‐bridged hydroxyl groups (3670 cm^−1^), whereas *t‐*ZrO_2_ favoured bi‐ (3735 cm^−1^) and tri‐bridged (3670 cm^−1^) species.^[60]^ The finding that bi‐bridged hydroxyls are unique to *t‐*ZrO_2_ whereas tri‐bridged hydroxyls form on both phases is in agreement with DRIFTS of the present samples (Figure S4), which reveal bi‐bridged (μ_2_‐Zr_2_OH) groups at 3737 and 3719 cm^−1^ dominate on *t*‐ZrO_2_ and tri‐bridged (μ_3_‐Zr_3_OH) at 3693 cm^−1^ for *m‐*ZrO_2_. The latter band is stronger for the mixed phase ZrO_2_ formed by calcining Zr(OH)_4_ at 400 °C. Terminal OH groups were not observed for any samples, which may reflect their highly crystalline nature and previous reports that low‐index crystal facets disfavour terminal Zr‐OH formation.[^61^, ^62^] Tri‐bridged groups can form on monoclinic zirconia when oxygen atoms in molecularly adsorbed water coordinate to Lewis acid sites to form [Zr^4+^‐OH_2_] centres;^[62]^ the appearance of strong μ_3_‐Zr_3_OH bands for *m‐*ZrO_2_ thus indicates significant Lewis acidity. Molecular modelling by Cerrato et al. considered how coordinatively unsaturated (cus) anions and cations on polar, low index zirconia facets restructure on reacting with molecular water. Dissociative adsorption, Zr^n+^_cus_O^2−^_cus_+H_2_O→[Zr^n+^OH^−^]^(n−1)^+OH^−^, results in hydroxylated surfaces, whereas for molecular adsorption, Zr^n+^_cus_O^2−^_cus_+H_2_O→[Zr^n+^OH_2_]O^2−^_cus_ OH^−^, the exposed anion remains coordinatively unsaturated. The former process is favoured over the (111) surface of *m‐*ZrO_2_ for which the Zr^n+^_cus_O^2−^_cus_ separation >2 Å, and yields terminal Zr‐OH and μ_3_‐Zr_3_OH species.^[62]^ Formation of tri‐ or bi‐bridged hydroxyls is largely dictated by the O^2−^ coordination in zirconia facets;^[63]^ for *t*‐ZrO_2_ the appearance of bi‐ and tri‐coordinated hydroxyls is consistent with preferential exposure of the (111) facets,^[64]^ as predicted.^[65]^

**Figure 4:**
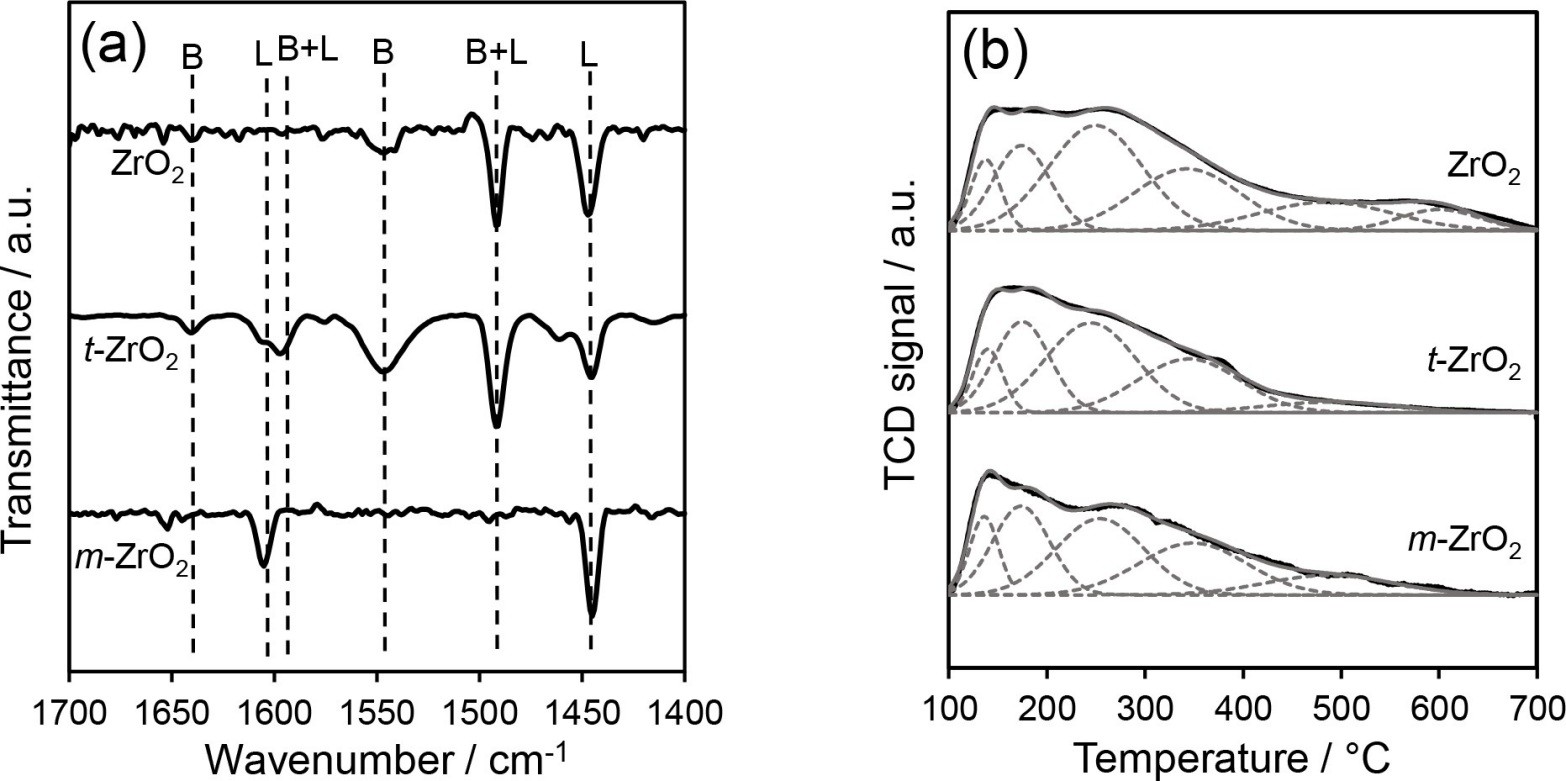
(a) Pyridine DRIFT spectra and (b) deconvoluted NH_3_‐TPD profiles of ZrO_2_, *t*‐ and *m*‐ZrO_2_ nanoparticles.

As outlined above, surface hydroxylation is expected to impact the surface acidity of zirconia, with the presence of Brønsted and Lewis acid sites examined by DRIFTS analysis of chemisorbed pyridine (Figure 4a). *m‐*ZrO_2_ exhibited bands at 1445 and 1602 cm^−1^ attributed to exclusive Lewis acidity, whereas *t‐*ZrO_2_ exhibited additional bands at 1545 and 1640 cm^−1^ attributed to pyridinium ions and hence Brønsted acidity.[^53^, ^66^] Bands at 1490 and 1597 cm^−1^ are common to Brønsted and Lewis sites.^[67]^ ZrO_2_ exhibited a similar spectrum to *t*‐ZrO_2_ (albeit weaker due to the low surface area of calcined nanoparticles) and thus mixed Brønsted and Lewis acidity, but with a lower Brønsted:Lewis ratio than the latter due to the presence of some monoclinic phase. These findings are in agreement with previous studies on ZrO_2_ polymorphs,^[68]^ and show that zirconia surface acidity depends on the crystalline phase and consequent surface reactivity to water.

Stoichiometric tetragonal (111) and monoclinic ($\bar{1}$ 11) surfaces are predicted to be the most stable for zirconia,^[65]^ however, calculations on relaxed surfaces of ZrO_2_ nanoparticles reveal that surface energies follow the order (101)<(001)<(100)<(111) for *t‐*ZrO_2_ and ($\bar{1}$ 11)<(011)<(001) for *m‐*ZrO_2_. Inspection of the surface terminations of tetragonal zirconia (Table S3) reveals μ_2_‐Zr_2_O bridging oxygen species dominate the (111) surface, μ_3_‐Zr_3_O the (100) and (001) surfaces, μ_4_‐Zr_4_O the (101) and (111) (Table S4). Assuming μ_3_‐ and μ_2_‐ bridging oxygens have the potential to form hydroxyls, the observation of μ_3_‐Zr_3_OH and μ_2_‐Zr_2_OH bands for *t‐*ZrO_2_ (Figure S4) suggests that (100), (001) and (111) facets dominate (in agreement with literature^[58]^). A similar analysis for monoclinic zirconia reveals μ_2_‐Zr_2_O dominates the (001) surface, μ_3_‐Zr_3_O the (011) and (111) surfaces, and μ_4_‐Zr_4_O the ($\bar{1}$ 11) surface. Since the μ_3_‐Zr_2_OH band dominates the DRIFTS spectrum of *m‐*ZrO_2_, we assume that these nanoparticles are dominated by (011) and (111) facets. The Brønsted acid strength of such bridging hydroxyls depends on the local electronic environment of Zr^4+^ cations in the bridges and presence of surface defects. This is analogous to how Brønsted acid sites form in metal‐organic frameworks (MOFs) wherein protic ligands (e. g., water) coordinate in bridging conformations between Lewis acid sites,^[69]^ with deprotonation of coordinated water spheres over Zr‐oxo bridges producing Brønsted acid sites.^[70]^ Scheme S1 shows the proposed stable facets for *m‐*ZrO_2_ and *t‐*ZrO_2_ based on the DFT calculations of Piskorz et al.,[^57^, ^58^] with low coordination Zr^4+^ sites and metal oxo bridges (identified in Table S4) giving rise to Lewis and Brønsted sites respectively. The smaller size of *t‐*ZrO_2_ nanocrystals (versus *m‐*ZrO_2_) is anticipated to create more low coordination sites analogous to those proposed responsible for Brønsted acid site formation at the nodes of Zr‐containing MOFs.^[70]^

The density and strength of acid and base sites were measured by temperature programmed desorption (TPD) using NH_3_ and CO_2_ as probe molecules, respectively. The *m*‐ZrO_2_ and *t*‐ZrO_2_ possessed comparable basicity, with that of ZrO_2_ approximately four times greater (Table 1 and Figure S5). The desorption envelope of the CO_2_TPD can be consistently fit across all three catalysts using five components with peak maxima <200 °C, between 200–400 °C, and >400 °C, respectively assigned to weak, medium, and strong base sites (Figure S5). Although all base strengths were observed for all catalysts, *t*‐ZrO_2_ was dominated by medium strength sites (200–450 °C). Weak base sites correspond to CO_2_ bound to basic hydroxyls as bicarbonate (HCO_3_^−)^ species which desorbs <200 °C; the mid‐strength species desorbing 200–400 °C correspond to bidentate carbonate (*b*‐CO_3_^2−^) bound to Zr^4+^‐O^2−^ acid–base pairs while the strongest species desorbing >400 °C are attributed to polydentate carbonate (*p*‐CO_3_^2−^) species coordinated to closely spaced coordinatively unsaturated Zr^4+^ centres or monodentate O^2−^ bound CO_3_^2−^ species (*m*‐CO_3_^2−^) shown in Scheme S2.^[71]^ *m*‐ZrO_2_ is reported to favour HCO_3_^−^, *m*‐CO_3_^2−^ and *b*‐CO_3_^2−^ species, while *t*‐ZrO_2_ favours *b*‐CO_3_^2−^ and *p*‐CO_3_^2−^ in agreement with Table S5.[^60^, ^72^] The intense feature at 300–400 °C for *t*‐ZrO_2_ is attributed to *m*‐CO_3_^2−^ and *p*‐CO_3_^2−^ species, suggesting a high proportion of coordinatively unsaturated Zr^4+^ and O^2−^ sites (which dominate the edges and corners of small nanoparticles). NH_3_ TPD profiles of all three samples revealed weak, medium and strong acid sites, defined as those desorbing ammonia <200 °C, between 200–400 °C, and >400 °C, respectively.^[73]^ The medium acid strength desorption ~350 °C is assigned to NH_3_ adsorbed on lower coordinated Zr^4+^ cations.^[74]^ The strong acid desorption (peak maximum ~500 °C) is tentatively assigned to NH_3_ released from Brønsted acid sites.^[75]^ Acid loadings were quantified by integrating desorption peaks fitted by Gaussian functions (using common peak widths and temperatures, Figure 4b). ZrO_2_ and *t*‐ZrO_2_ exhibited comparable weak and medium acid site loadings (Table 1), with ZrO_2_ and *m*‐ZrO_2_ exhibiting comparable strong acid site loadings. Acid site loadings were subsequently used in conjunction with integrated pyridine bands to quantify the loading of Brønsted and Lewis sites on the parent ZrO_2_ materials, which are shown in Table S6. This confirms the Brønsted acid site density is highest over *t‐*ZrO_2_ at 2 μmol m^−2^_,_ while *m*‐ZrO_2_ has the highest Lewis acid density of 5 μmol m^−2^.

### Catalytic Reactivity

#### Glucose Isomerisation

**Figure 5:**
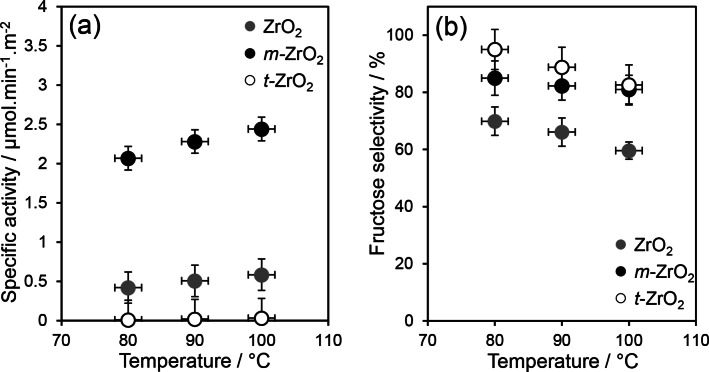
Impact of ZrO_2_ phase on glucose isomerisation to fructose: (a) specific activity for glucose conversion and b) fructose selectivity. Reaction conditions: 6 h batch; 100 °C; 200 mg catalyst; 0.56 mmol glucose, 20 mL water.

The preceding three materials were subsequently studied for batchwise glucose conversion after 6 h reaction at 80, 90 and 100 °C (Figure 5 and S6–7). It is extremely challenging to prepare pure phase zirconia nanoparticles with similar surface areas and thus the surface area normalised rates of glucose isomerisation (Figure 5a) were used to determine intrinsic activity of the mixed phase ZrO_2_, (Lewis acidic) *m*‐ZrO_2_ and (Brønsted acidic) *t*‐ZrO_2_ samples. At all temperatures, the surface area normalised, specific activity over *m*‐ZrO_2_ was >10× that over *t*‐ZrO_2_ (Figure 5a) demonstrating that glucose isomerisation is highly sensitive to the zirconia phase/facet. Despite containing 86 wt % monoclinic zirconia and possessing four times the total acid loading, the mixed phase ZrO_2_ catalyst was four times less active than *m*‐ZrO_2_. Furthermore, while mixed phase ZrO_2_ achieved the highest absolute glucose conversion (42 % versus 28 % for *m*‐ZrO_2_, Table S7) at 100 °C due to its significantly greater surface area (Table 1), the fructose selectivity was poor (Figure 5b) in comparison with *m‐*ZrO_2_ demonstrating the importance of controlling acid type. Although possessing the highest acid loading (0.43 mmol g^−1^), *t‐*ZrO_2_ showed the lowest conversion (12 %) evidencing the majority of the (Brønsted, μ_2_‐Zr_2_OH) acid sites present cannot activate glucose under our conditions. As expected, glucose conversion exhibited a modest increase with reaction temperature (Figure S6), whereas selectivity to fructose (similar for both pure phase zirconias) decreased due to glucose condensation and humin formation[^17^, ^76^] (from ~90 % at 80 °C–~82 % at 100 °C, Figure 5b). Only trace HMF (<1.5 μmol) was observed over the pure phase catalysts under any condition. Thermodynamics dictates a maximum fructose yield of 57 % at 100 °C, approximately double that of the highest yield of 25 % observed (Table S7).^[25]^ Nanoparticulate *m*‐ZrO_2_ is thus a highly selective and stable catalyst for glucose isomerisation to fructose, which compares favourably to homogeneous NaOH (60 % selectivity at 50 % conversion and 90 °C,^[77]^ and some of the best reported solid bases (e. g. Mg doped NaY zeolites) which suffer from Na and Mg leaching,^[78]^ or Lewis acidic Sn‐doped zeolites (58 % selectivity at 50 % conversion and 120 °C,^[23]^ which rapidly deactivate in flow due to Sn leaching.^[79]^ However, as glucose isomerisation is rate‐limiting for the overall cascade production of HMF, a higher area *m*‐ZrO_2_ catalyst would undoubtedly be beneficial but remains synthetically elusive.

Turnover frequencies (TOFs) calculated per acid site were highest for *m*‐ZrO_2_ (33 h^−1^) followed by the mixed phase ZrO_2_ (10 h^−1^) and *t*‐ZrO_2_ (6 h^−1^), evidencing glucose isomerisation is structure sensitive and favoured by the weak Lewis acid sites (including penta‐coordinated Zr^4+^, Table S4) that dominate the *m‐*ZrO_2_ surface (Figure 4a and Table 1).[^57^, ^58^] These values compare favourably to literature values for magnesium doped ZrO_2_ (TOF=7 h^−1^).^[80]^ The mechanism of glucose→fructose isomerisation is widely accepted to be initiated by base and/or Lewis acid sites and thus base sites (Table S5) could potentially contribute to the high activity of the mixed phase ZrO_2_ (Figure S6). However, inorganic base catalysts also promote side reactions,^[81]^ and are less selective than Lewis acid sites that catalyse isomerisation via intramolecular hydride shifts rather than proton transfers.^[23]^ This may account for the lower fructose selectivity of the mixed phase ZrO_2_. Base sites promote elimination of −OH^−^ from enediolate intermediates formed in the glucose‐fructose isomerisation step,^[22]^ yielding unstable deoxy‐α‐dicarbonyl compounds that rapidly condense with sugars (via aldol reactions) to form oligomers. Base sites can also catalyse undesired retro‐aldol reactions of glucose and fructose to form short chain glycoaldehyde and D‐erythrose (or dihydroxyacetone and D‐glyceraldehyde) products respectively,[^81^, ^82^] consistent with our observation that the mixed phase ZrO_2_ rich in base sites (0.41 mmol g^−1^) was the least selective to fructose (Figure 5b and S8). In the context of aqueous‐phase glucose isomerisation, a previous study^[83]^ has also highlighted competitive adsorption between water and glucose for Lewis acid sites, with hydrophilic materials hindering glucose adsorption resulting in zero‐order kinetics with respect to [glucose]. In addition to differences in Lewis/Brønsted character, the higher TOF of *m*‐ZrO_2_ versus *t*‐ZrO_2_ and ZrO_2_ may also arise from a stronger affinity of the former for glucose, i. e., *m*‐ZrO_2_ is less hydrophilic. This possibility was investigated by TGA and NMR relaxation measurements (see later).

#### Fructose Dehydration

**Figure 6:**
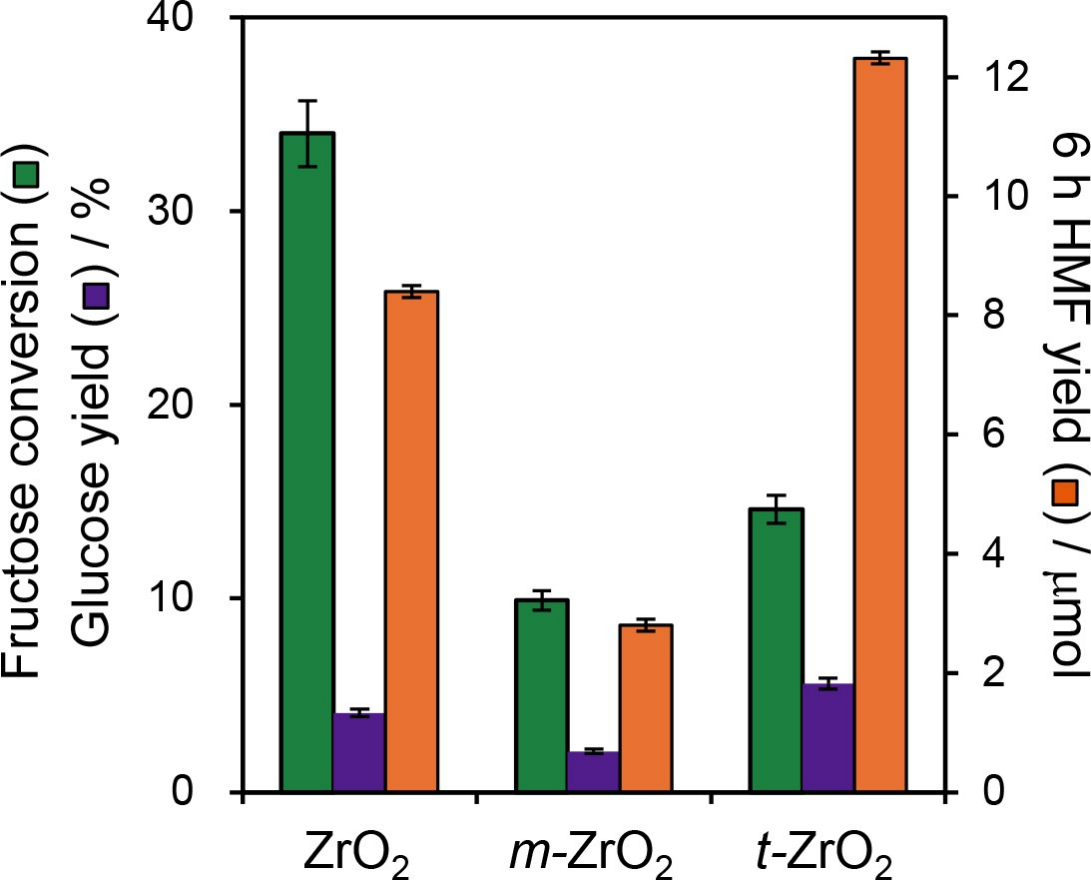
Fructose dehydration to 5‐HMF over ZrO_2_, *m*‐ZrO_2_, *t*‐ZrO_2_ catalysts. Reaction conditions: 6 h batch; 0.56 mmol fructose, 20 mL water at 100 °C using 200 mg of catalyst.

The efficacy of nanoparticulate zirconias for the second step in the cascade, fructose dehydration to HMF, was also explored (Figure 6). All catalysts were active for fructose dehydration, however, *t*‐ZrO_2_ proved superior for HMF production than either ZrO_2_ or *m*‐ZrO_2_, attributed to the high density of Brønsted acid sites in the former. Lewis acidic *m*‐ZrO_2_ exhibited similar HMF production to a catalyst‐free control experiment (0.4 % yield vs 0.17 %). Despite exhibiting a high activity for fructose conversion, the mixed phase ZrO_2_ yielded 35 % less HMF than *t*‐ZrO_2_, indicating that the base sites of ZrO_2_ promoted undesired side reactions.

#### Cascade Glucose Conversion to HMF

**Figure 7:**
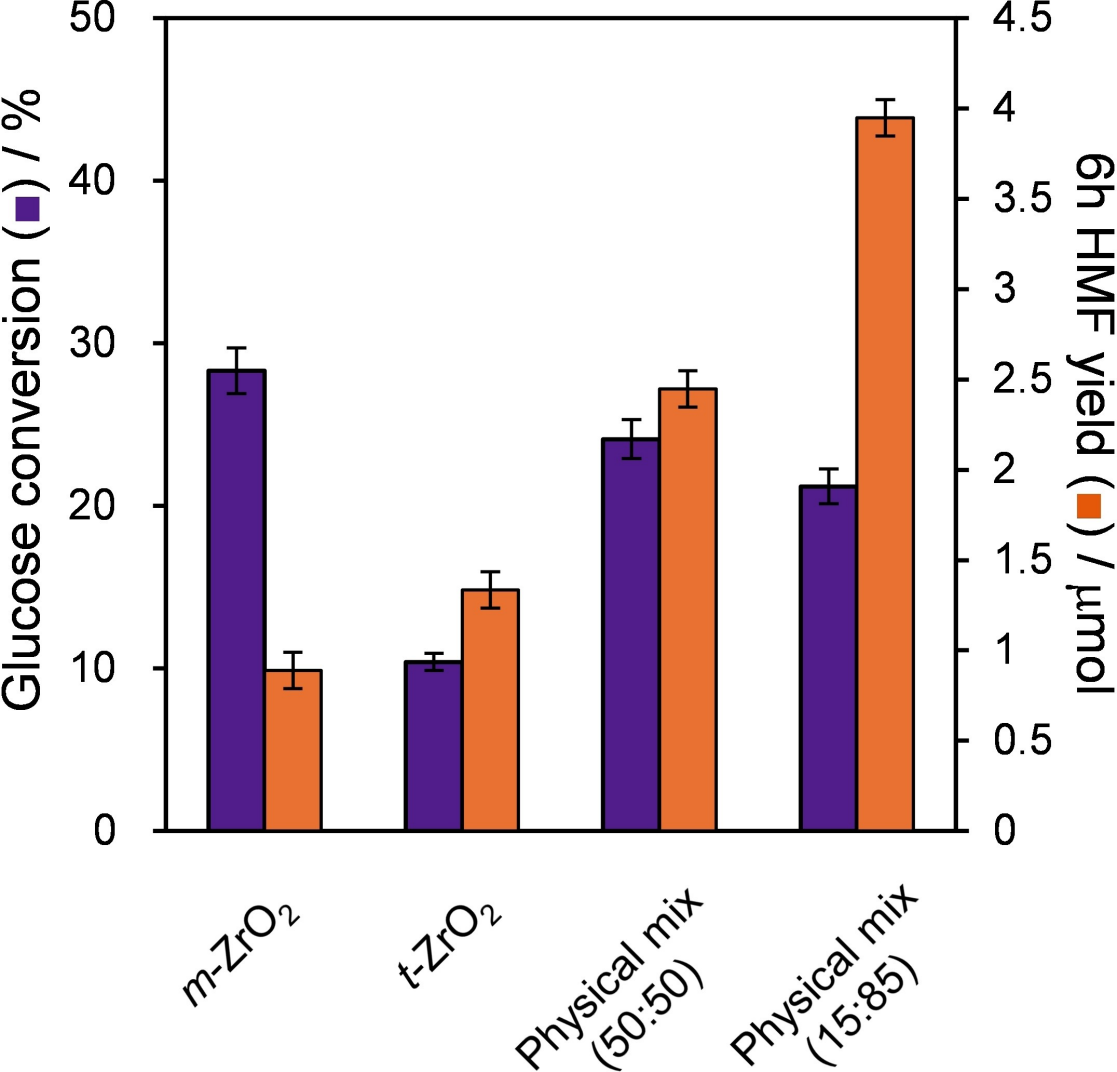
Glucose conversion to 5‐HMF over ZrO_2_, *m*‐ZrO_2_, *t*‐ZrO_2_ and [*m*‐ZrO_2_+*t*‐ZrO_2_] physical mixtures. Reaction conditions: 6 h batch; 0.56 mmol fructose, 20 mL water at 100 °C using 200 mg of a single catalyst or physical mixture. Physical mixture (50 : 50) uses 100 mg each of of *m‐* and *t‐*ZrO_2_; Physical mixture (15 : 85) uses 30 mg of *m‐*ZrO_2_ and 170 mg *t‐*ZrO_2_.

**Scheme 2:**
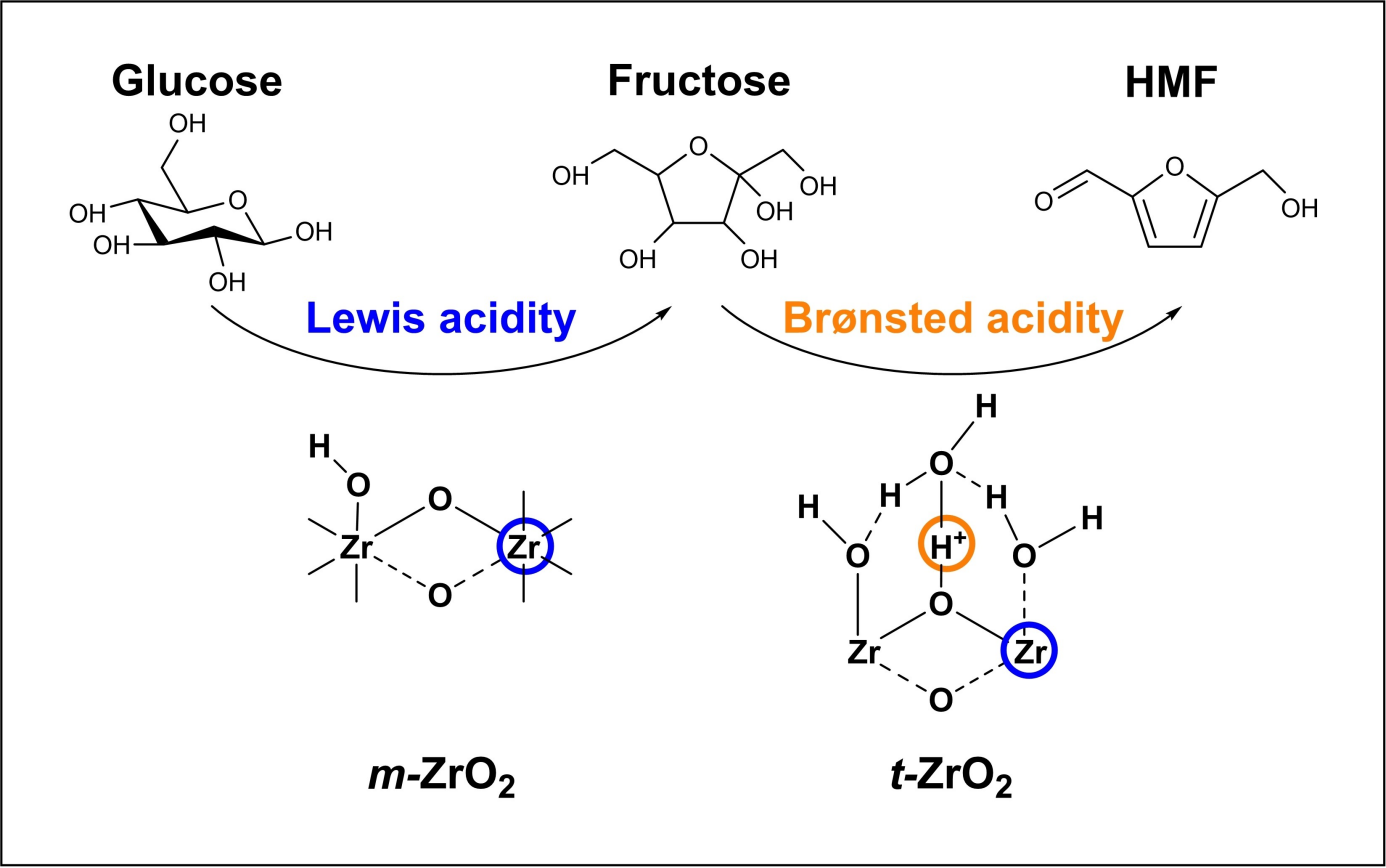
Lewis acid sites at undercoordinated Zr^4+^ sites (blue circle) on *m*‐ZrO_2_ surfaces promote glucose isomerisation to fructose, while Brønsted acid sites formed by polarisation of bridging hydroxyls (orange circle) with water chemisorbed at undercoordinated Zr^4+^ sites on *tNMR*‐ZrO_2_ promote fructose dehydration to 5‐HMF.

The presence of Lewis and Brønsted acid sites at the surfaces of different zirconia phases should lend itself to the one‐pot cascade conversion of glucose to HMF. To test this hypothesis, the performance of a physical mixture of *m*‐ZrO_2_:*t*‐ZrO_2_ (in a 1 : 1 mass ratio) with each pure phase was compared against both pure phases in a batch reactor (Figure 7). The Lewis acidic *m*‐ZrO_2_ exhibited modest glucose conversion (~27 %) but only trace HMF (<0.9 μmol), and the Brønsted acidic *t*‐ZrO_2_ showed poor glucose conversion but a higher HMF yield (~1.3 μmol), in accordance with their individual reactivities for glucose isomerisation and fructose dehydration (Figure 5–6 and Table S7). However, in combination, the 1 : 1 physical mixture of *m*‐ZrO_2_:*t*‐ZrO_2_ delivers both modest glucose conversion and a significant yield of HMF (2.5 μmol), evidencing synergy between the two phases. Varying the mass ratios (Figure S9), identified the optimum physical mixture as 15 wt % *m‐*ZrO_2_:85 wt % *t‐*ZrO_2_ equating to 4 μmol HMF (albeit only 0.7 % yield). Glucose conversion was first order with respect to the *m‐*ZrO_2_ loading (Figure S10a), and HMF production first order with respect to *t‐*ZrO_2_ loading between 15 and 50 % (Figure S10b), consistent with the proposed models for: (i) Lewis acid catalysed glucose isomerisation to fructose (over *m‐*ZrO_2_); and (ii) Brønsted acid catalysed fructose dehydration to HMF (over *t‐*ZrO_2_) illustrated in Scheme 2.

Unsurprisingly, HMF productivity falls at high *t‐*ZrO_2_ loadings, becoming limited by the low concentration of reactively‐formed fructose attainable over low *m‐*ZrO_2_ loadings, and indeed we find a linear relationship between B : L ratio (for different physical mixtures of *m*‐ and *t*‐ZrO_2_ in batchwise glucose conversion) (Figure S11). These reaction kinetics also suggest reactions were free from mass transport limitations. Note that the HMF yield for this optimal physical mixture is still significantly lower than that attained over pure *t‐*ZrO_2_ from fructose (12 μmol, Figure 6), in part due to the low fructose yield from glucose (~25 % over *m‐*ZrO_2_) and hence undesired, competitive adsorption of unreacted glucose at *t‐*ZrO_2_ sites.^[84]^ Although our HMF yields are lower than many literature reports,[^85^, ^86^] such high yields typically require biphasic systems or ionic liquids (normally 30–60 vol%), and/or higher temperature (140–200 °C) in pressurised reactors, often with inorganic promoters such as NaCl.^[87]^ Quantitative kinetic studies of intrinsic catalyst reactivity necessitate deliberate operation at modest conversion in a simple solvent to eliminate mass‐transport limitations, but despite the mild reaction conditions, the performance of our (15 : 85) *m:t* ZrO_2_ physical mixture compares very favourably with literature aqueous phase studies (Table S8a). Fructose dehydration to HMF is by definition challenging to perform in water due to the reverse hydrolysis reaction. Previous studies indicate that calcined zirconia (exposing both *m‐*ZrO_2_ and *t‐*ZrO_2_) can effectively catalyse glucose conversion to HMF in batch, but was performed in a biphasic DMSO:H_2_O system at 170 °C,^[35]^ wherein fructose dehydration can be directly catalysed by DMSO (thereby increasing HMF yields).^[88]^ The present observations confirm that appropriate mixtures of *m‐*ZrO_2_ and *t‐*ZrO_2_ phases, devoid of dopants, can catalyse the cascade at only 100 °C in water (aligning with the principles of green chemistry^[89]^).

**Figure 8:**
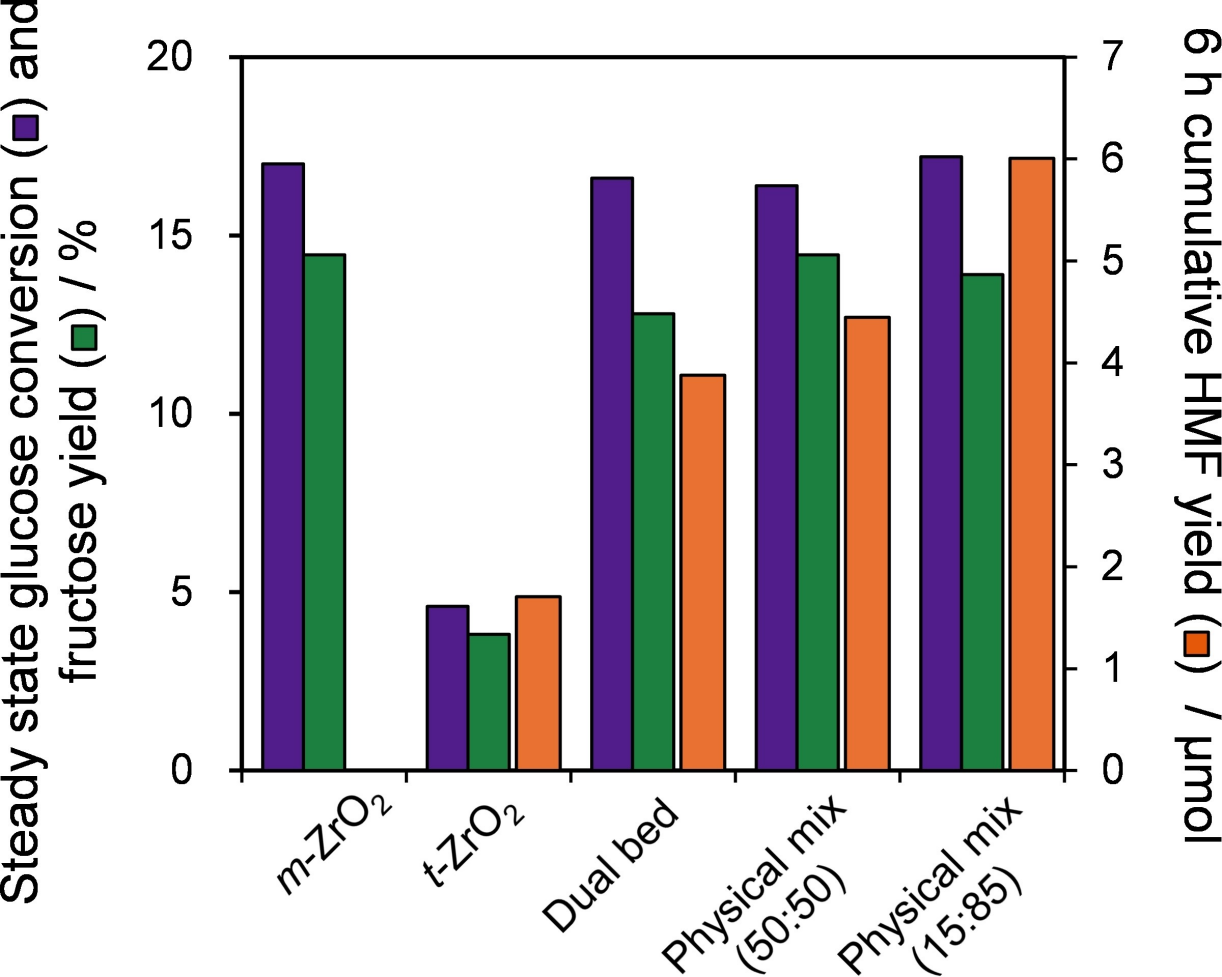
Continuous flow glucose conversion over *m*‐ and *t*‐ZrO_2_ and different catalyst bed configurations of *m‐* and *t‐*ZrO_2_. Conditions: τ=50 min total for all catalyst beds, with 28 mM glucose solution, 100 °C, with a duration of 6 h. Pure phase 200 mg of *m‐* or *t‐*ZrO_2_; dual bed 200 mg of catalyst (100 mg *m‐*ZrO_2_+100 mg *t*‐ZrO_2_); physical mixture (50 : 50) 100 mg each of *m‐* and *t‐*ZrO_2_; physical mixture (15 : 85) 30 mg of *m‐*ZrO_2_ and 170 mg *t‐*ZrO_2_.

To further assess the potential of nanoparticulate ZrO_2_ in the cascade conversion of glucose to 5‐HMF, the reaction was explored in a packed bed reactor under continuous flow, which offers numerous process advantages.[^90^, ^91^] To our knowledge, the zirconia catalysed conversion of glucose to HMF in continuous flow has not been previously reported. The flow conversion of glucose at 100 °C was performed with a cumulative 6 h substrate feed equivalent to that present in batch reactions to enable direct comparison of performance (Figure 8). As anticipated, *m‐*ZrO_2_ was effective for glucose isomerisation, with a mean conversion ~17 %, somewhat lower than attained in batch (28 %) reflecting the shorter residence time of 50 min in flow. Fructose was the sole product (~14 % yield); the absence of even trace HMF suggests that fructose dehydration kinetics were simply too slow compared to the residence time for this Lewis acid catalyst. Consistent with Figure 7, *t‐*ZrO_2_ exhibited poor activity for glucose conversion (<5 %) but its Brønsted acidity catalysed some fructose dehydration to HMF (~1.5 μmols). In efforts to improve rates of glucose conversion and fructose dehydration, the pure phase zirconias were subsequently combined in various configurations. First, a dual bed configuration was studied, comprising contiguous beds of 100 mg *m*‐ZrO_2_ followed by 100 mg *t*‐ZrO_2_, with the aim of supplying a higher concentration of reactively‐formed fructose from the first bed for dehydration to HMF over the second bed. The results confirmed that hypothesis, approximately doubling the HMF yield compared to *t*‐ZrO_2_ (~4 μmols) and demonstrating that glucose isomerisation is likely rate‐limiting for HMF production under these conditions.^[17]^ To ascertain whether the spatial arrangement of zirconia phases mattered, a 50 : 50 physical mixture of both catalysts in a single packed bed was also examined; this configuration offered a slight increase in HMF yield, indicating that dispersing *m‐*ZrO_2_ in *t*‐ZrO_2_ may help maintain a uniform level of fructose (from glucose isomerisation) throughout the mixed bed. Since the thermodynamic maximum fructose yield from glucose isomerisation at 100 °C is 57 %, some unreacted glucose will always contact the *t*‐ZrO_2_ bed in both dual and mixed bed configurations. Batch experiments in Figure 7 show that a 15 : 85 (*m*‐ZrO_2_:*t*‐ZrO_2_) ratio was optimal for HMF production, and hence this composition was explored as a physical mixture in a single reactor bed in flow. Despite decreasing the amount of Lewis acidic monoclinic phase, glucose conversion was unchanged, however, the additional Brønsted acidic tetragonal phase was now able to produce ~6 μmols HMF. This evidences: (i) glucose isomerisation had reached equilibrium in our system; and (ii) a strong cooperativity between Lewis and Brønsted acid sites placed in close proximity. The lower yields of HMF attained from glucose versus fructose (Figure 6, >12 μmols) may stem from competitive adsorption of unreacted glucose over the *t‐*ZrO_2_ catalyst. Reports suggest that glucose preferentially binds to sorbent surfaces (e. g. chromatographic columns) when in excess relative to fructose (and vice versa).^[84]^ Under our conditions, only ~20 % of the initial glucose is converted to fructose, and hence fructose adsorption/dehydration over *t*‐ZrO_2_ is likely inhibited by the excess glucose.

The enhancement of HMF production using the physical mixture relative to that from *t*‐ZrO_2_ was calculated according to Equation 5.

(5) $$\mathrm{Relative}\;\mathrm{enhancement}\;(\%)=\frac{Total\;HMF\;yield\;of\;physical\;mixture\;}{Total\;HMF\;yield\;of\;t-{ZrO}_{2}}\;\times \;100$$

**Figure 9:**
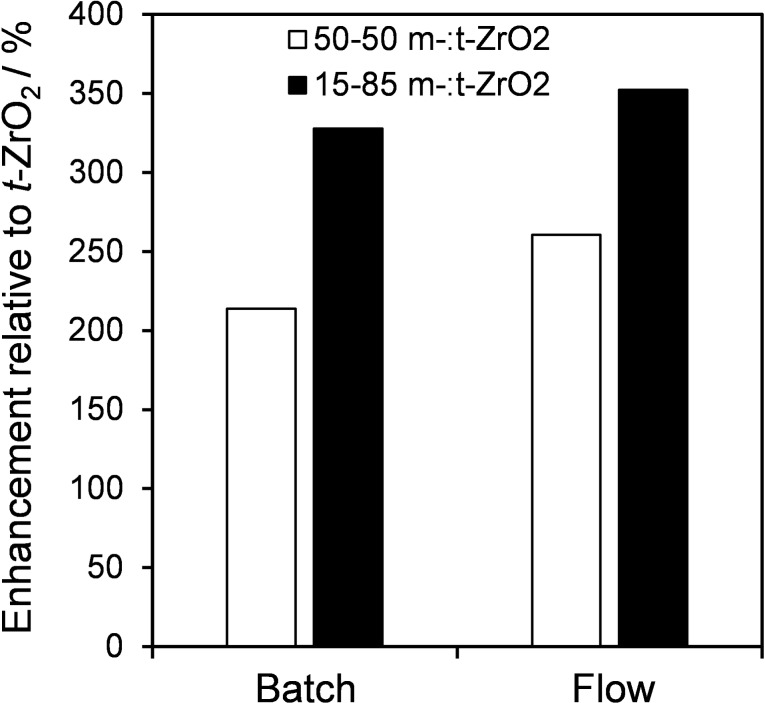
HMF yield enhancement for the cascade conversion of glucose to HMF over physical mixtures of *m‐*ZrO_2_ and *t‐*ZrO_2_ relative to *t‐*ZrO_2_ in batch and continuous flow after 6 h at 100 °C.

A comparison of the two physical mixtures of *m*‐ZrO_2_:*t*‐ZrO_2_ in batch and flow reveals that both offer greatly enhanced HMF yields compared to a comparable mass of *t‐*ZrO_2_ alone (Figure 9), a consequence of the aforementioned cooperativity in catalysing the two‐step cascade in Scheme 2. Continuous flow operation, in which both zirconia phases are maintained in proximity, was also superior to batchwise operation wherein cooperativity between the dispersed phases will be less effective. Time‐on‐stream measurements revealed the optimal 15 : 85 mass ratio of *m‐*ZrO_2_:*t‐*ZrO_2_ in a physical mixture maintained a constant 18 % glucose conversion and cumulative HMF production of 6 μmols and 18 μmols over 6 h and 20 h respectively with negligible deactivation (Figure S12a and 12b). Increasing the glucose concentration from 28 mM–280 mM at 100 °C also enhanced the cumulative HMF yield achieved over the *m‐*ZrO_2_:*t‐*ZrO_2_ 15 : 85 physical mixture from 6–15 μmols (Figure S13). To explore whether further increases in HMF yield were possible, continuous flow reaction was also explored at 150 °C for 6 h (Figure S14), which increased glucose conversion to 58 %. Corresponding yields of fructose and HMF were 22 % and 49.9 μmol respectively, representing cumulative yield of 268 μmol over 6 h and a 50‐fold increase in HMF productivity versus reaction at 100 °C (Figure S12a). However, higher reaction temperatures also promote side reactions, lowering the carbon mass balance to ~50 %. These observations do however compare favourably with previous studies of continuous flow HMF production under aqueous conditions using any solid catalyst (Table S8b), highlighting the potential for further process optimisation of these mixed‐phase catalyst beds in conjunction with biphasic solvent mixtures for the continuous extraction of HMF.

#### NMR Relaxation of Molecular Adsorbates

**Figure 10:**
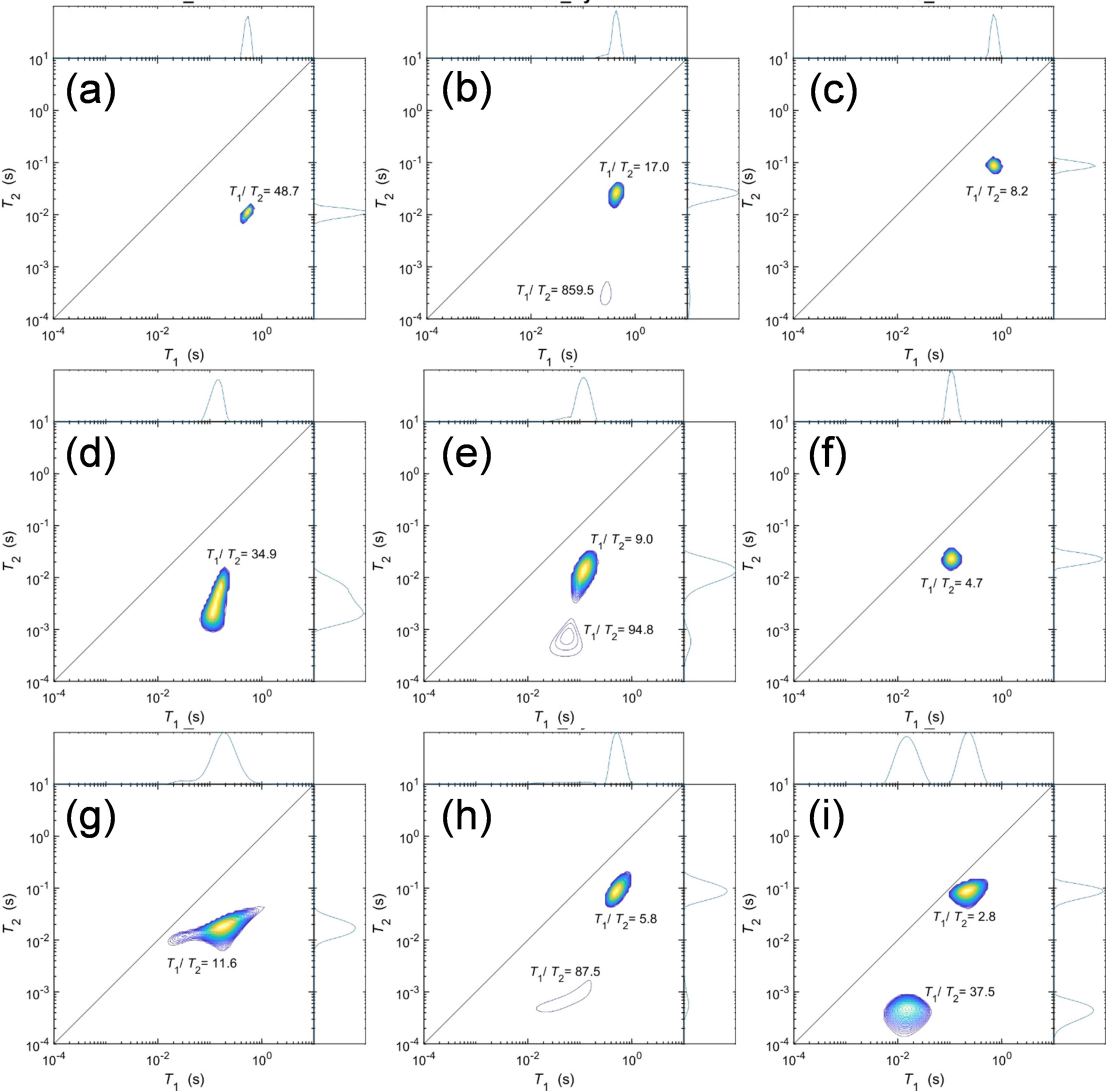
*NMR T*_1_‐*T*_2_ relaxation correlation plots of ZrO_2_ for (a) water, (b) pyridine and (c) THF; *t*‐ZrO_2_ for (d) water, (e) pyridine and (f) THF; *m*‐ZrO_2_ for (g) water, (h) pyridine, and (i) THF.

In a heterogeneous catalytic reaction, reactant adsorption is critical to activating functional groups and directing surface reactions to the desired products. Many such reactions employ solvents in large excess to the reactant, and hence solvent interactions with catalyst surfaces can also modulate reactivity through, e. g. blocking of active sites or (de)stabilising reactive intermediates. NMR relaxation measurements offer a quantitative probe of the strength of molecular interactions with catalyst surfaces, and in this work were explored for a range of probe molecules (water, pyridine, THF, and n‐octane as a hydrophobic reference) of differing hydrophilicity and basicity (Figure 10), wherein the ratio of the longitudinal to transverse relaxation times (*T*_1_/*T*_2_) indicates the strength of surface interaction.^[92]^ The pulse sequence is detailed in Figure S15.

For mixed phase ZrO_2_, the *T*_1_/*T*_2_ value was 48.7 with water compared to 34.3 and 11.8 in *t*‐ZrO_2_ and *m*‐ZrO_2_, respectively (Table 2). The significant disparity for the monoclinic phase, suggesting a weaker interaction between water molecules and surface hydroxyls of m‐ZrO_2_, consistent with the lower OH density determined by TGA (Table 1), and consistent with previous work reported on zeolitic materials.^[93]^ The strong affinity of ZrO_2_ and *t‐*ZrO_2_ for water may account for their poor activity for glucose isomerisation; strongly adsorbed water likely hinders reactant adsorption with any available Lewis acid sites as previously observed for zeolites.^[94]^ The higher *T*_1_/*T*_2_ observed for pyridine over ZrO_2_ and *t‐*ZrO_2_ is consistent with their higher total acid density and Brønsted acidity observed by DRIFTS and NH_3_ TPD (Figure 4). When probing Lewis acid sites using tetrahydrofuran (THF), *m*‐ZrO_2_ shows two distinct environments: one with a relatively low *T*_1_/*T*_2_ value of 2.8 (weak Lewis acid) and another with significantly higher *T*_1_/*T*_2_ value of 37.5 (strong Lewis acid) in comparison to that of 8.2 for ZrO_2_ and 4.8 for *t*‐ZrO_2_. This observation is in good agreement with NH_3_‐TPD that shows *m*‐ZrO_2_ has a higher proportion of weak and medium strength Lewis sites than the other catalysts (Table 1). This variation in Lewis acidity is likely the underlying cause of the superior glucose isomerisation activity noted in *m*‐ZrO_2_ compared to the other catalysts.

**Table 2:** *NMR T*_1_/*T*_2_ ratio values forZrO_2_ catalysts using different probe molecules.

Catalyst	*T*_1_/*T*_2_ relaxation value of different probe molecules			
	H_2_O	Pyridine	THF	n‐Octane
ZrO_2_	48.7	17.1	9.9	8.2
*t*‐ZrO_2_	34.3	8.9	4.8	3.3
*m*‐ZrO_2_	11.8	5.8	2.8, 37.5	6.9

#### Post‐Reaction Catalyst Characterisation

**Figure 11:**
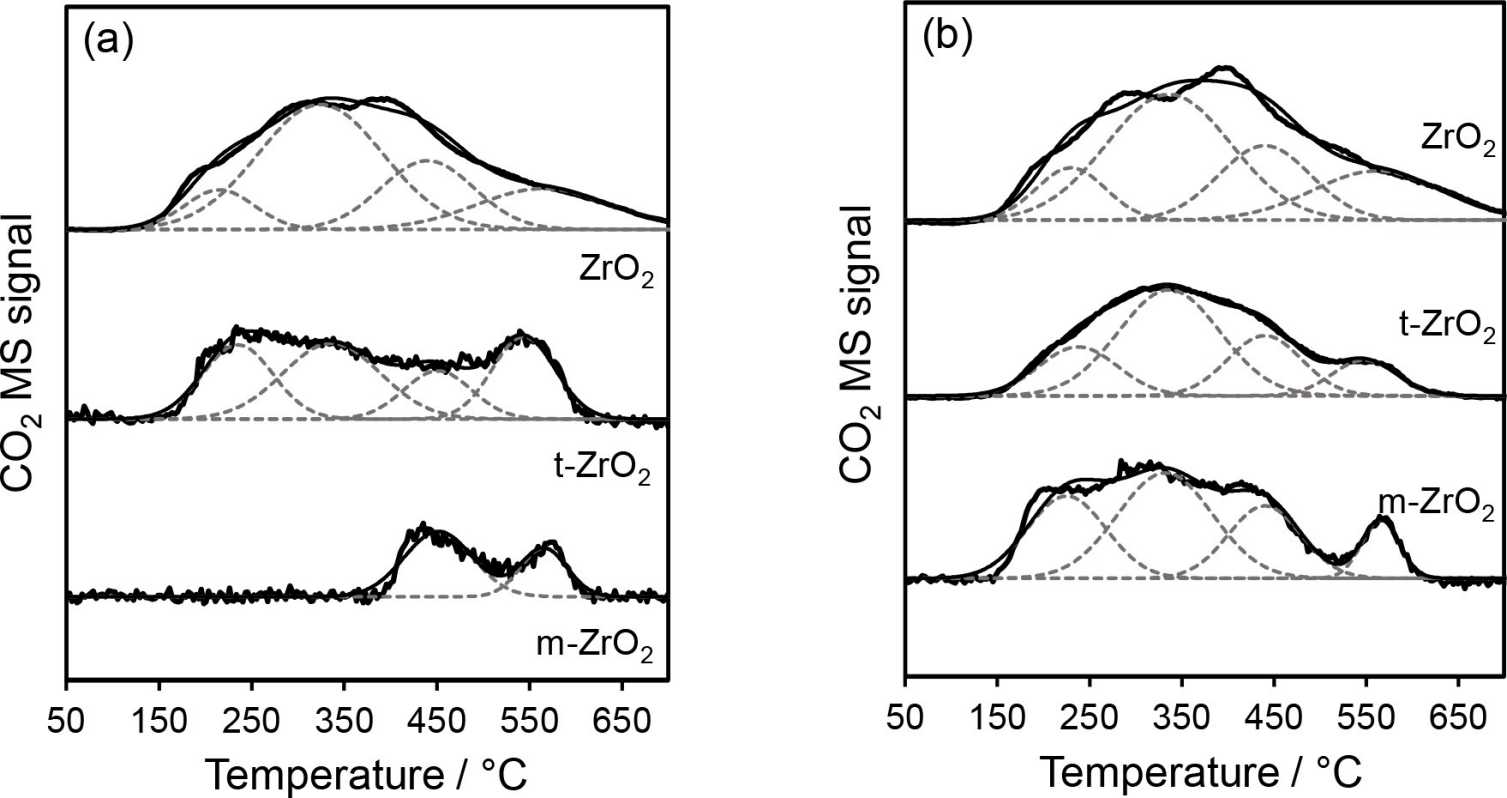
CO_2_ (44 amu) desorption signal during TPO of catalysts after batch reaction of (a) glucose and (b) fructose. Conditions: Samples were heated from 35–800 °C, at a ramping rate of 10 °C min^−1^ under a 10 mL min^−1^ flow of compressed air.

Temperature programmed oxidation (TPO) was performed to characterise carbonaceous residues present on catalyst surfaces post‐reaction. For the two‐step cascade conversion of glucose to HMF, surface carbon increased in the sequence of *m*‐ZrO_2_<*t*‐ZrO_2_<ZrO_2_, whereas carbon accumulation following reaction with fructose increased in the order of *m*‐ZrO_2_<ZrO_2_<*t*‐ZrO_2_ (Figure S16). The amount of carbon species accumulated on *t*‐ZrO_2_ was greater following reactions with fructose rather than glucose, consistent with stronger adsorption (reaction) of pure fructose over *t*‐ZrO_2_ versus *t*‐ZrO_2_, and hence higher HMF productivity over the former. CO_2_ desorptions at ~443 °C and 569 °C (Figure 11) are attributed to amorphous coke and graphitic carbon, respectively, while desorptions ~232 °C and 340 °C are indicative of strongly adsorbed hydrocarbon products. Overall, *m*‐ZrO_2_ exhibits greater resistance to coking and deactivation by organic residue adsorption. Only small amounts of surface carbon accumulate during glucose isomerisation to fructose over *m‐*ZrO_2_ compared to direct reaction with fructose, suggesting that the presence of glucose inhibits undesirable side reactions. In light of these measurements, catalyst reactivation of the 15 : 85 *m*‐:*t*‐ZrO_2_ physical mixture was explored by 700 °C calcination, with subsequent recycling in batch revealing excellent stability (Figure S17) with only ~25 % decrease after five batchwise reactions (in part due to catalyst losses). Glucose conversion slightly increased in the second reaction cycle, accompanied by a slight decrease in fructose yield, which likely reflects partial loss of the *t*‐ZrO_2_ phase (or crystallite sintering) following repeated thermal regeneration. Computational modelling of the (competitive) adsorption of ketose and aldose forms of sugars^[15]^ over *m‐* and *t‐*ZrO_2_ surfaces would help provide valuable insight into the reaction mechanism and potential pathways to by‐products.

## Conclusions

Aqueous phase production of HMF from glucose, under mild conditions and employing a low‐cost catalyst, would offer a sustainable chemical manufacturing route to this sought after bio‐derived platform chemical. The dual Brønsted/Lewis acid character of Earth abundant zirconia was exploited in this study for the direct, aqueous phase conversion of glucose to HMF through a catalytic cascade. Pure phase monoclinic (*m*−) and tetragonal (*t*−) zirconia nanoparticles were prepared by hydrothermal synthesis from a common zirconyl nitrate precursor using either urea or a Pluronic surfactant, respectively. *m*‐ZrO_2_ formed as ~30 nm crystallites with a low surface area (~20 m^2^ g^−1^) that predominantly exposed (011) and (111) facets exhibiting Lewis acidity, whereas *t*‐ZrO_2_ formed as ~8 nm crystallites (~150 m^2^ g^−1^) exposing (100), (001) and (111) facets exhibiting Brønsted acidity. Lewis acidic *m*‐ZrO_2_ showed moderate activity but high (~81 %) selectivity for glucose isomerisation to fructose at 100 °C but was unable to dehydrate fructose to HMF. In contrast, *t*‐ZrO_2_ was essentially inert towards glucose but active for fructose dehydration at 100 °C. NMR relaxation measurements indicate *m*‐ZrO_2_ was weakly hydrophilic and hence likely able to stabilise a higher proportion of Lewis acid sites required for glucose adsorption and isomerisation to fructose, whereas *t*‐ZrO_2_ was strongly hydrophilic and hence well‐suited to Brønsted acid catalysed fructose dehydration (even in water). Physical mixtures of both pure phases enabled cooperative catalysis in batch and (packed bed) continuous flow reactors, with a 15 : 85 mass ratio of *m‐*ZrO_2_:*t‐*ZrO_2_ optimal. A contiguous, dual bed configuration of *m‐*ZrO_2_ followed by *t‐*ZrO_2_ was not advantageous compared to a single mixed bed, evidencing the importance of rapid transport and adsorption of reactively‐formed fructose at Brønsted acid sites to minimise undesired side reactions (or fructose interconversion back to glucose). Continuous flow operation mitigates by‐product poisoning of active sites, by removing them from the reaction zone, but presents a challenge for efficient glucose consumption throughout the reactor bed. Continuous downstream product separation and recycling of unreacted glucose, and the synthesis of higher area pure phase zirconias (e. g. by dispersing over porous silicas) offer opportunities to increase HMF productivity.

## Conflict of Interests

The authors declare no conflict of interest.

1

## Supporting information

As a service to our authors and readers, this journal provides supporting information supplied by the authors. Such materials are peer reviewed and may be re‐organized for online delivery, but are not copy‐edited or typeset. Technical support issues arising from supporting information (other than missing files) should be addressed to the authors.

Supporting Information

## Data Availability

The data that support the findings of this study are available from the corresponding author upon reasonable request.
