# Supplementary material for: Phase Effects in Zirconia Catalysed Glucose Conversion to 5‐(Hydroxymethyl)furfural
Source: ChemSusChem. 2024 Nov 21;18(4):e202401494. doi: 10.1002/cssc.202401494 (PMC11826133; doi:10.1002/cssc.202401494)
Supplement: Supplementary file 1 — Supporting Information [file CSSC-18-e202401494-s001.pdf]

ChemSusChem

Supporting Information

Phase Effects in Zirconia Catalysed Glucose Conversion to 5-(Hydroxymethyl)furfural

Yang Liu, Luke Forster, Aristarchos Mavridis, Andrea Merenda, Mohamed Ahmed, Carmine D′Agostino, Muxina Konarova, Aaron Seeber, Enrico Della Gaspera,* Adam F. Lee,* and Karen Wilson*

Supporting information

Phase Effects In Zirconia Catalysed Glucose Conversion To 5-Hydroxy Methylfurfural

Yang Liu,[1] Luke Forster,[2] Aristarchos Mavridis,[2] Andrea Merenda,[3,4] Mohamed Ahmed,[5] Carmine D'Agostino,[2,6] Muxina Konarova,[5] Aaron Seeber,[7] Enrico Della Gaspera,*[1] Adam F. Lee,*[8] Karen Wilson*[8]

[1]School of Science, RMIT University, Melbourne VIC 3000, Australia

[2]Department of Chemical Engineering, The University of Manchester, The University Of Manchester, Manchester M13 9PL, UK

[3]Australian Research Council Research Hub for Nutrients in a Circular Economy, Centre for Technology in Water and Wastewater, School of Civil and Environmental Engineering, University of Technology Sydney, NSW 2007, Australia

[4]Faculty of Engineering and Information Technology, University of Technology Sydney, Ultimo NSW 2007, Australia

[5]School of Chemical Engineering, The University of Queensland, Brisbane, QLD 4072, Australia

[6]Dipartimento di Ingegneria Civile, Chimica, Ambientale e dei Materiali (DICAM), Alma Mater Studiorum - Università di Bologna, 40131 Bologna, Italy

[7]CSIRO Manufacturing, 71 Normandy Rd, Clayton North, Melbourne, VIC 3168, Australia

[8]Centre for Catalysis and Clean Energy, Griffith University, Gold Coast QLD 4222, Australia

*Corresponding Authors: enrico.dellagaspera@rmit.edu.au; adam.lee@griffith.edu.au; karen.wilson6@griffith.edu.au

S1 Material characterisation

Table S1. Rietveld refinement data of zirconia from XRD patterns

Catalyst	Lattice structure	Phase weight percentage / %	Crystallite domain size	Lattice parameter			
				a / Å	b / Å	c / Å	β / °
ZrO_2	Tetragonal	14.3(6)	8(2)	3.62(2)	-	5.13(6)	-
	Monoclinic	85.7(6)	8.6(3)	5.152(1)	5.203(1)	5.320(1)	99.16(1)
t-ZrO_2	Tetragonal	100	6.7(2)	3.5916(4)	-	5.180(1)	-
	Monoclinic	-	-	-	-	-	-
m-ZrO_2	Tetragonal	0.9(5)	n/a*	-	-	-	-
	Monoclinic	99.1(5)	17.9(4)	5.1478(7)	5.2062(7)	5.3172(7)	99.218(5)

*: Crystallite size not calculatable due to low peak intensities and significant peak overlap.

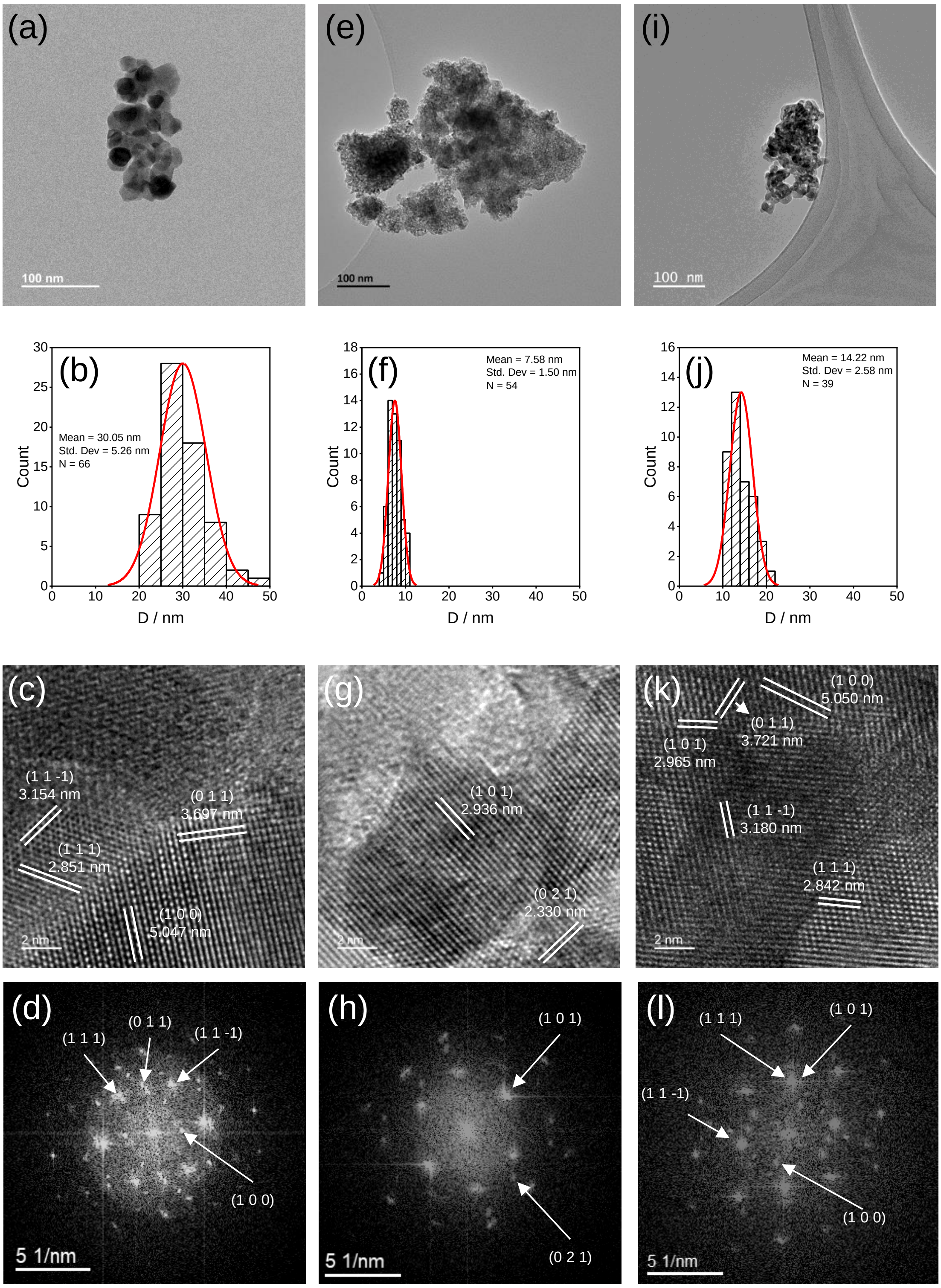

Figure S1. TEM, particle size distribution histogram, HRTEM, fast-Fourier-transform (FFT) images of zirconia nanoparticles: *m*-ZrO_2 (a-d), *t*-ZrO_2 (e-h) and mixed phase ZrO_2 (i-l) where the (111), (100) and (11-1) are from *m-FFT* ZrO_2 and (101) from *t*-ZrO_2 components.

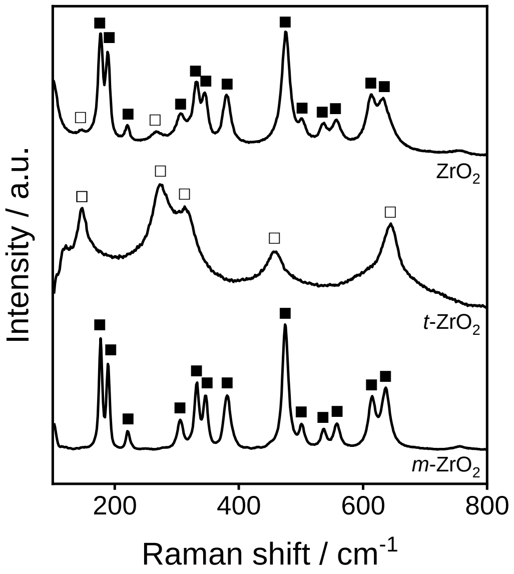

Figure S2. Raman spectra of mixed-phase ZrO_2, *m*- and *t*-ZrO_2. ■: monoclinic phase; □: tetragonal phase.

Table S2 Raman-active phonon modes of zirconia[1]

**Symmetry of vibrational mode for ZrO_2 phase**	**Assignment of Raman mode / cm^{-1}**
**Tetragonal**[2]	
B_{1g}	146 (O-Zr-O), 312 (O-O)
E_g	274, 459, 645 (Zr-O)
**Monoclinic**[3]	
A_g	189 (Zr-Zr), 346 (Zr-O), 382, 475, 559, 637 (O-O)
B_g	305 (Zr-O), 331 (Zr-Zr), 382, 501, 536, 615 (O-O)
A_g+B_g	176 (Zr-Zr)

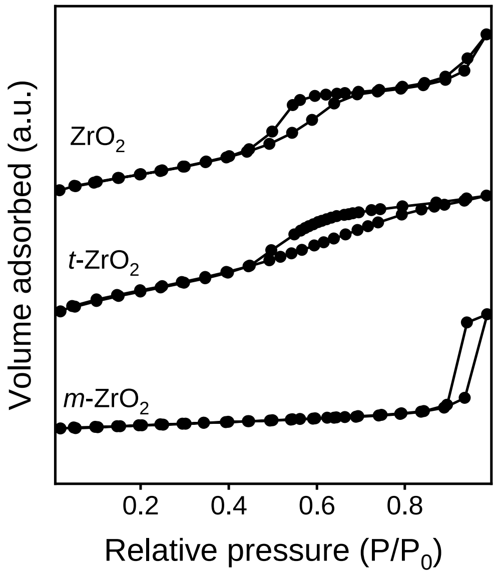

Figure S3. N_2 adsorption-desorption isotherm of ZrO_2, *t*- and *m*-ZrO_2

Table S3. Pore volume and average pore diameter of ZrO_2, *t*- and *m*-ZrO_2

Catalyst	Pore volume / cm^3g^{-1}	Average pore diameter / nm
ZrO_2	0.25	4.3
t-ZrO_2	0.32	3.8
m-ZrO_2	0.14	27.4

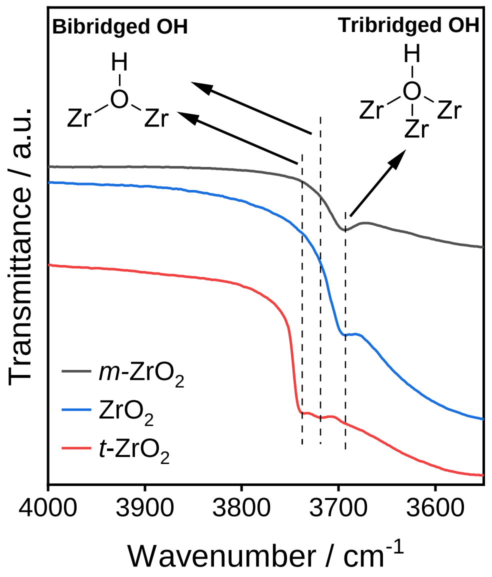

Figure S4. DRIFTS spectra of surface OH vibrations and corresponding surface configuration on ZrO_2.

Table S4. The fraction of different types of Zr^{4+} and O^{2-} coordination in the unit cell of exposed stable facets of *m*- or *t*-ZrO_2. (adapted from refs [4, 5]).

		***m*-ZrO_2 facet**			
		**(-1 1 1)**	**(1 1 1)**	**(0 1 1)**	**(0 0 1)**
**O^{2-} in unit cell**		7	6	8	8
**Fraction of O^{2-} in coordination environment**	Bi-bridge	0.14	0.33	0.25	0.5
	Tri-bridge	0.29	0.50	0.75	0.25
	Tetragonal	0.57	0.17	-	0.25
**Zr^{4+} in unit cell**		4	4	4	4
**Fraction of Zr^{4+} with coordination number**	5 coord			0.5	0.5
	6 coord	0.75	1	0.5	0.6
	7 coord	0.25			
		***t*-ZrO_2 facet**			
		**(1 0 1)**	**(1 1 1)**	**(1 0 0)**	**(0 0 1)**
**O^{2-} in unit cell**		12	12	8	8
**Fraction of O^{2-} in coordination environment**	Bi-bridge	-	0.5	-	0.33
	Tri-bridge	0.5	-	1	0.67
	Tetragonal	0.5	0.5	-	-
**Zr^{4+} in unit cell**		8	8	4	4
**Fraction of Zr^{4+} with coordination number**	5 coord		0.5		
	6 coord			1	1
	7 coord	1	0.5		

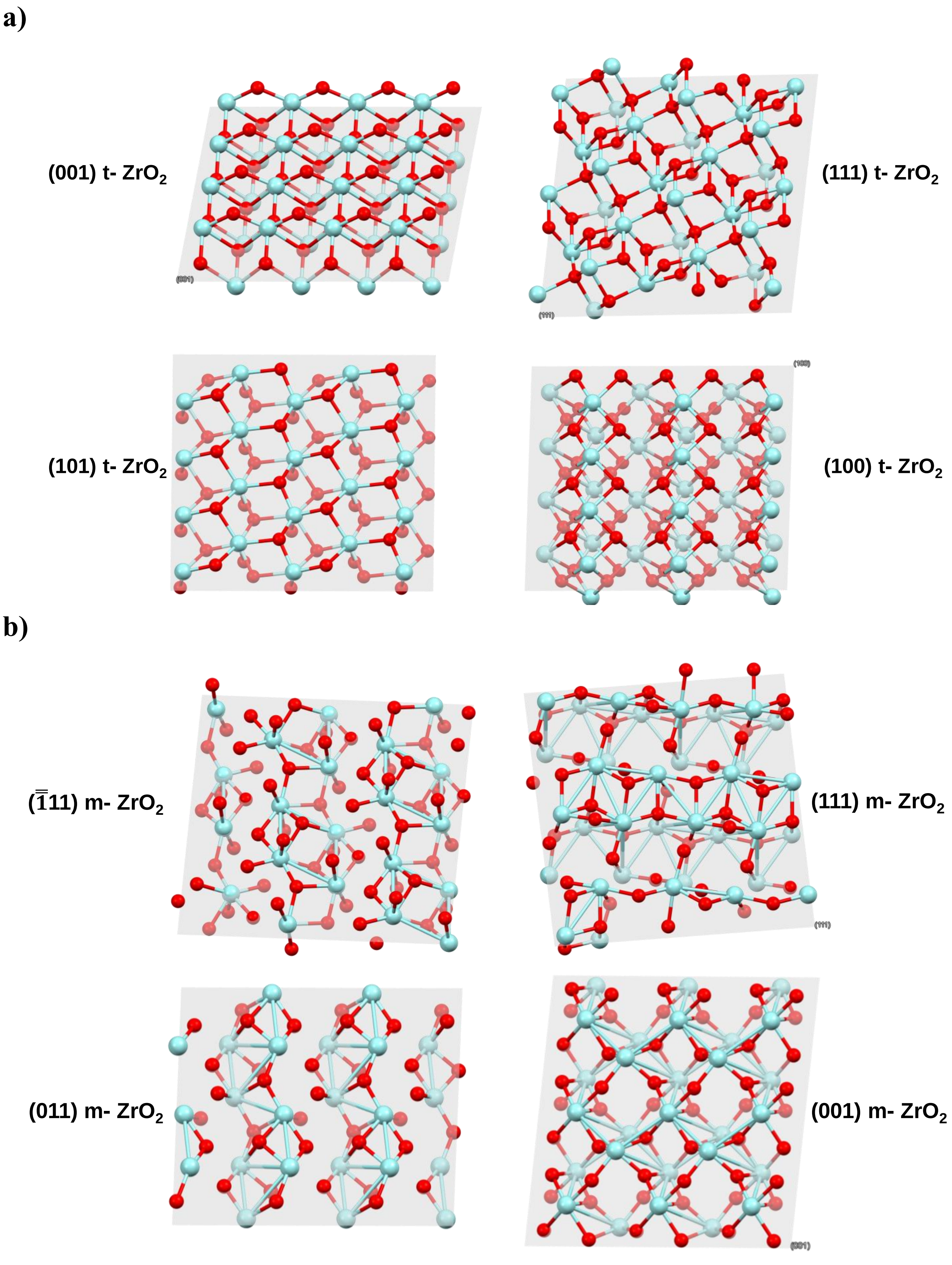

Scheme S1: Stable exposed surface facets of nanoparticulate (a) *t*-ZrO_2 and (b) *m*-ZrO_2 (as identified in references 4, 5).

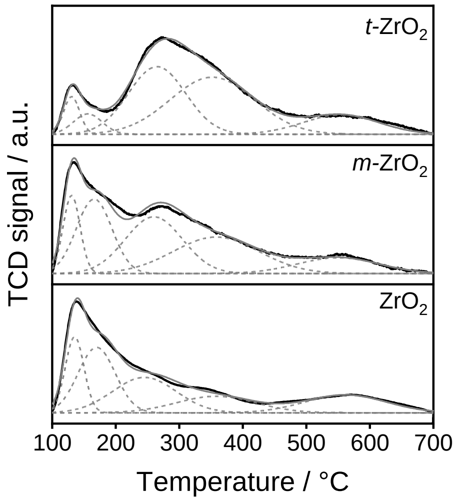

Figure S5. CO_2-TPD profiles of ZrO_2, *m*- and *t*-ZrO_2 nanoparticles. For clarity of presentation intensity, desorption signals for *t*- and *m*-ZrO_2 samples are multiplied five-fold.

Table S5. Base sites loadings determined by CO_2-TPD.

Catalyst	Base loading / mmol g^{-1}	Weak base loading[a] / µmol g^{-1}	Medium base loading[b] / µmol g^{-1}	Strong base loading[c] / µmol g^{-1}
ZrO_2	0.41	179	159	69
t-ZrO_2	0.11	11	83	15
m-ZrO_2	0.10	37	52	13

[a]: <200 °C; [b]: 200-400°C; [c]: >400 °C

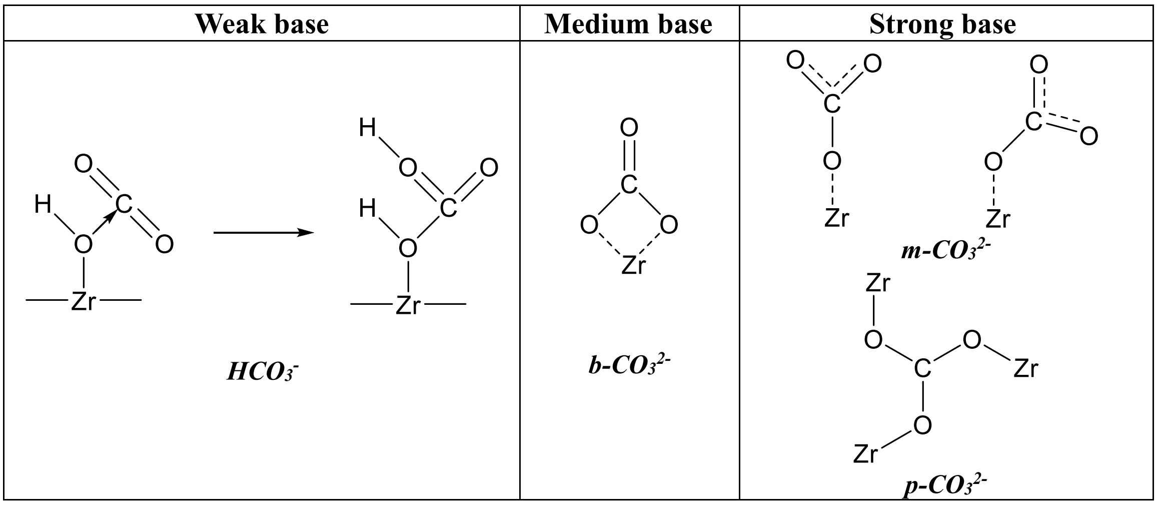

Scheme S2 Coordination environment of CO_2 at weak, medium and strong base sites of ZrO_2

Quantifiation of the pyridine IR spectra using [**Eq. S1**] below was performed using corrected IR band intensites and total acid site loadings from NH_3-TPD

$$\frac{L}{B} = \left(\frac{a_{Lpy}}{a_{Bpy}}\right)\left(\frac{1}{ECR}\right) \quad \text{(Eq. S1)}$$

Where a_{Lpy} and a_{Bpy} are the peak areas of the Lewis band at 1445 cm^{-1} and Brønsted band at 1545 cm^{-1}, respectively. ECR is the extinction coefficient ratio of coordinatively bonded pyridine (Lpy) and protonated pyridine (Bpy) as per the literature (*J. Catal.* **1999**, *183*, 45).[6]

Table S6. Acid loadings determined by pyridine IR and NH_3 titration.

Catalyst	Brønsted acid loading / mmol g^{-1}	Brønsted acid density / mmol m^{-2}	Lewis acid loading / mmol g^{-1}	Lewis acid density / mmol m^{-2}
ZrO_2	0.11	0.001	0.28	0.002
t-ZrO_2	0.25	0.002	0.18	0.001
m-ZrO_2	-		0.10	0.005

S2 Catalyst testing

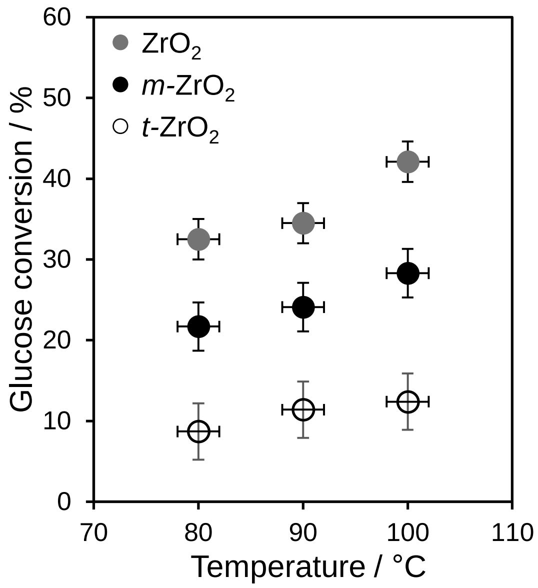

Figure S6. Absolute glucose conversion of ZrO_2, *t*- and *m*-ZrO_2 at 80, 90 and 100 °C. Reaction conditions: 6 h batch reaction; 200 mg catalyst; 0.56 mmol glucose, 20 mL water.

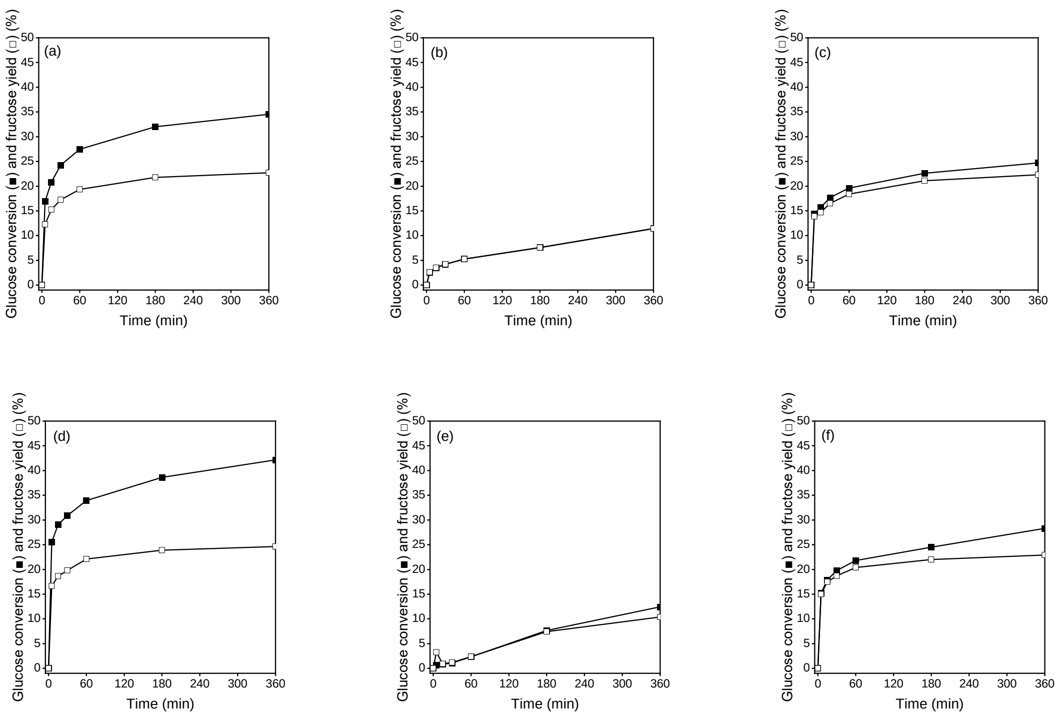

Figure S7. Glucose isomerisation reaction profiles of a) ZrO_2, b) *t*-ZrO_2 and c) *m*-ZrO_2 at 90 °C, d) ZrO_2; e) *t*-ZrO_2 and f) *m*-ZrO_2 at 100 °C Reaction conditions: 6 h batch reaction; 200 mg catalyst; 0.56 mmol glucose, 20 mL water.

Table S7. Conversion, yield and normalised glucose initial activity over zirconia catalysts.

Catalyst	Glucose conversion / %	Fructose selectivity / %	Fructose yield / %	Surface area / $m^2 \cdot g^{-1}$	Crystallite size / nm	Initial activity[a] / $\mu mol \cdot m^{-2} \cdot min^{-1}$	TOF[b] / h^{-1}
ZrO_2	42	59	25	127	8.0 (tetragonal)<br>8.6 (monoclinic)	0.53	10
m-ZrO_2	28	81	23	22	17.9	2.43	33
t-ZrO_2	12	83	10	153	6.7	0.29	6

[a]Reaction conditions: 6 h batch reaction; 100 °C; 200 mg catalyst; 0.56 mmol glucose, 20 mL water. [b]Normalised to acid site loading.

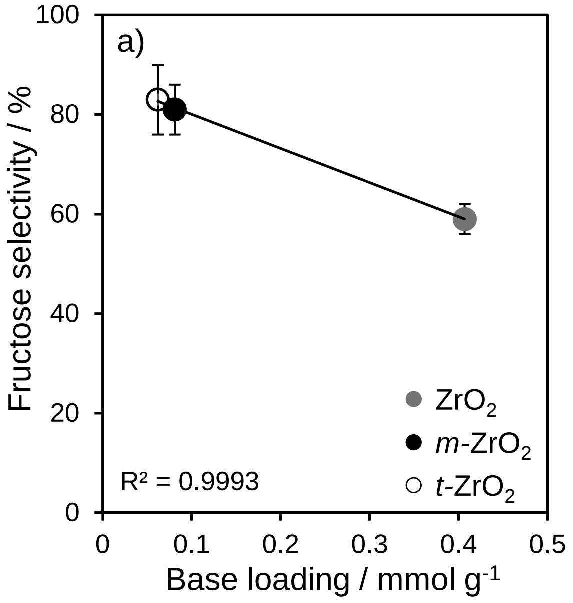

Figure S8. Correlation between a) fructose selectivity and base site loading under batch reaction conditions after 6 h.

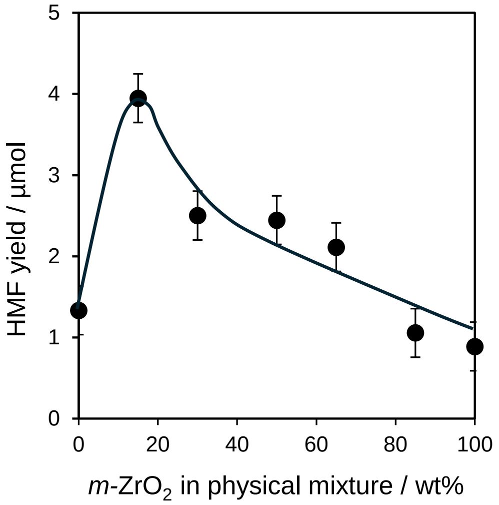

Figure S9. Impact of *m*-ZrO_2 content on HMF yield from glucose in batch using a physical mixture of *m*- and *t*-ZrO_2.

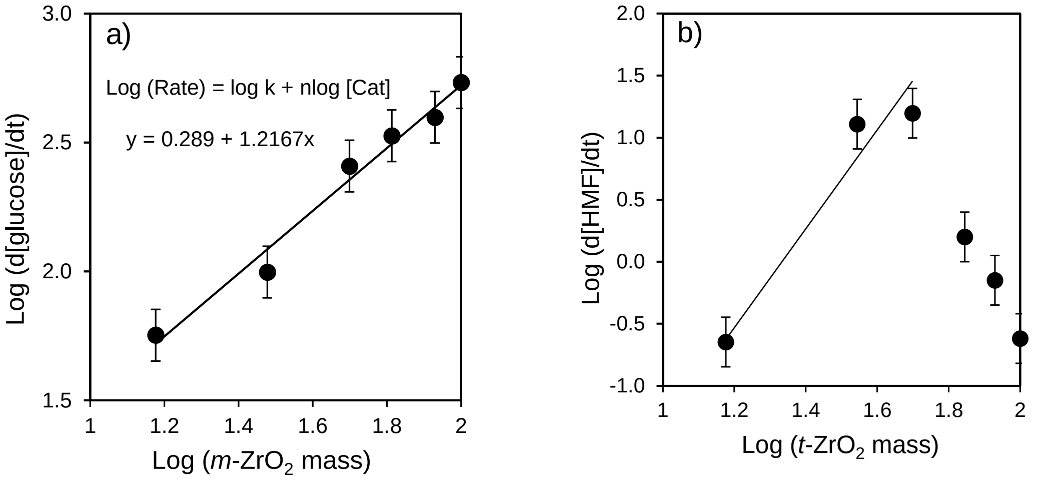

Figure S10. Effect of m-ZrO_2:t-ZrO_2 ratio on cascade glucose conversion in batch. a) log plot to verify that glucose isomerisation is first order with respect to [m-ZrO_2] and b) HMF yield is first order with respect to [t-ZrO_2] between 15 and 50 wt% t-ZrO_2. Deviation at the extremes is accounted for by mass transport limitations in fructose production for higher [*t*-ZrO_2].

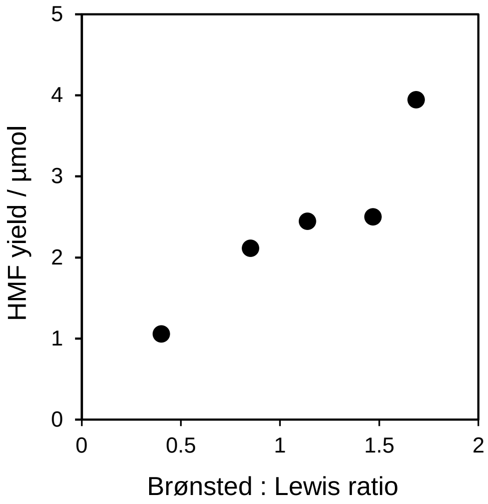

Figure S11. HMF yield as a function of Brønsted:Lewis acid ratio for glucose conversion in batch using a physical mixture of *m*- and *t*-ZrO_2.

Table S8a. Aqueous phase glucose conversion to HMF in batch in the absence of co-solvents.

Glucose conc^{n} /wt%	Catalyst	Temperature / °C	Reaction time	Conversion / %	Fructose yield / %	Fructose selectivity / %	HMF Yield / %	HMF Selectivity / %	Ref
0.5 wt%	Physical mixture of m- : t-ZrO_2 in 15:85 ratio	100	6 h	21.2	15.2	71.7	0.7	3.4	This work
0.5 wt%	$ZrOCl_2$	155	6 h	98.5	-	-	2.7	2.7	[7]*
0.5 wt%	m-W/ZrO_2	155	6 h	83.4	-	-	3.9	4.7	[7]*
10 wt%	$SnCl_4 \cdot 5H_2O$	110	1 h	90	0	0	-	-	[8]
10 wt%	Zeolite Ti-β	110	1.5 h	74	14	18.9	-	-	[8]
0.5 wt%	1ML-SZ/SBA15	100	6 h	3.3	2.9	86.4	0.5	13.6	[9]

**Pressurised batch reactors.*

Table S8b. Aqueous phase glucose conversion to HMF under continuous flow in the absence of co-solvents.

Glucose concentration	Solvent	Catalyst	Temperature / °C	Back Pressure (Bar)	Residence time / min	Glucose conversion / %	HMF Yield / %	HMF Selectivity / %	Ref
0.5 wt%	Water	*m*-:*t*-ZrO_2 in 15:85 mass ratio	150	6	50	58.3	7.6	13	This work
23 wt%	Water:n-BuOH (3:1)	TiO_2	180	138	3		6	-	[10]

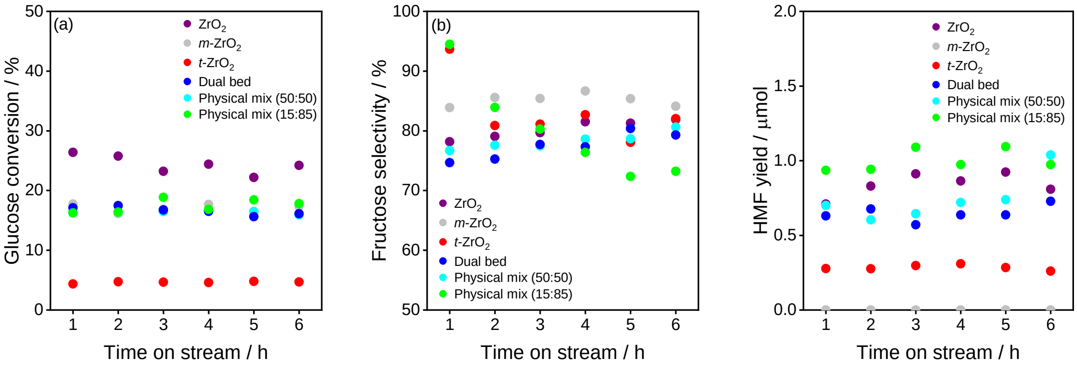

Figure S12a. Time-on-stream data of (a) glucose conversion, (b) fructose yield, and (c) HMF yield for the continuous flow glucose reaction. Continuous flow reaction conditions: τ = 50 min total for all catalyst beds, 100 °C; Single bed uses 100 mg of *m*- and *t*-ZrO_2, 200 mg of ZrO_2; Dual Bed uses 200 mg of catalyst (100 mg *m*-ZrO_2 followed by 100 mg t-ZrO_2); Physical mixture (50:50) uses 100 mg each of of *m*- and *t*-ZrO_2; Physical mixture (15:85) uses 30 mg of *m*-ZrO_2 and 170 mg *t*-ZrO_2.
and dual-bed and physical mixture); 28mM glucose in water, with a duration of 6 h.

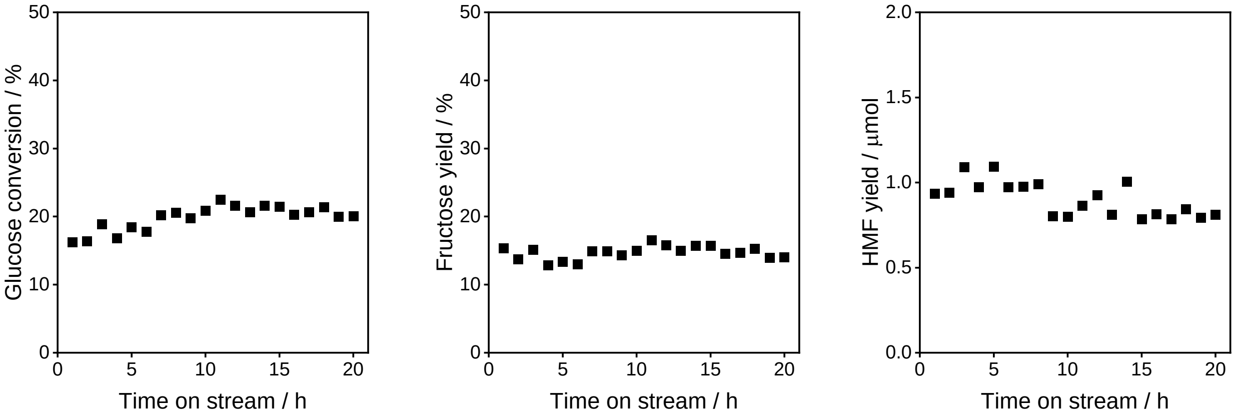

Figure S12b. Stability data for (a) glucose conversion, (b) fructose yield, and (c) HMF yield over 15:85 *m*:*t*-ZrO_2 physical mixture in continuous flow. Reaction conditions: τ = 50 min, 100 °C; 30:170 mg *m*-:*t*-ZrO_2; 28 mM glucose in water, with a duration of 20 h.

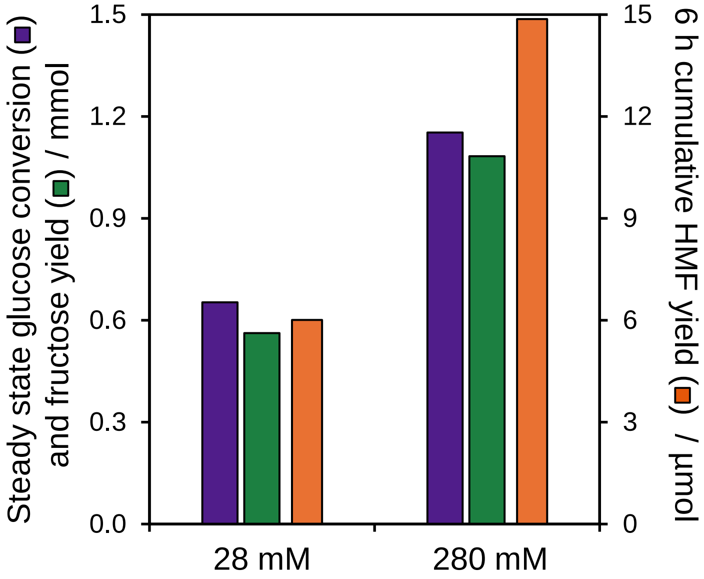

Figure S13. Steady state glucose conversion, fructose yield, and HMF yield for the continous flow cascade reaction over a 15:85 mass ratio of *m*-ZrO_2 and *t*-ZrO_2 (200 mg total). Reaction conditions: τ = 50 min total; 100 °C; 28mM glucose in water; 6 h.

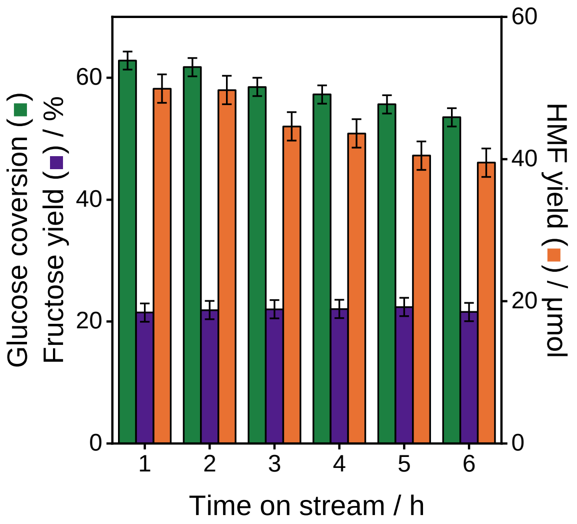

Figure S14. Glucose conversion, fructose yield, and HMF yield over over 15:85 m:t-ZrO_2 physical mixture in continuous flow. Reaction conditions: τ = 50 min, 150 °C; 30:170 mg of m-:t-ZrO_2; 28 mM glucose in water, 6 h.

S3 NMR relaxation of molecular adsorbates

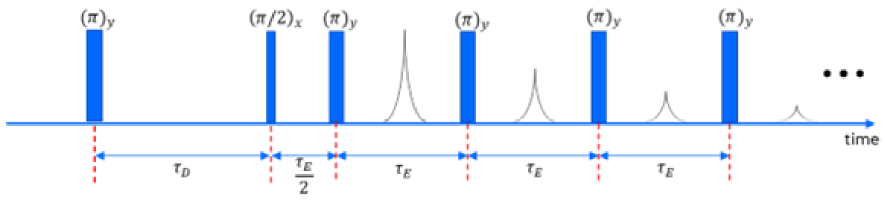

Figure S15. T_1-T_2 relaxation pulse sequence. The rectangular blocks represent electromagnetic pulses which "rotate" the net magnetization of the sample. The pulses are separated by predetermined time intervals, where τ_D is the delay time and τ_E is the echo time.

S4 Catalyst regeneration and reuse

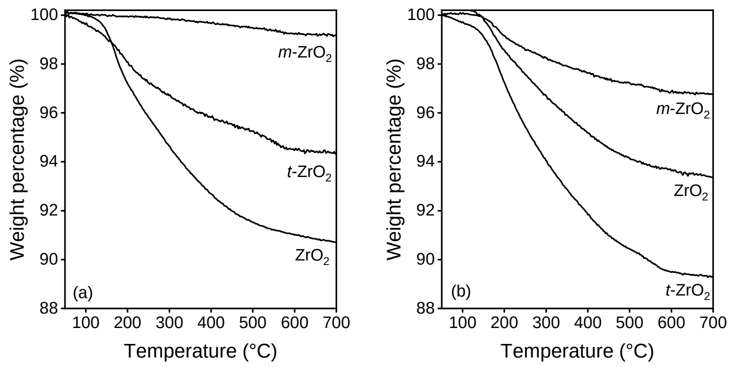

Figure S16. TGA profile of spent catalysts following reaction with (a) glucose or (b) fructose.

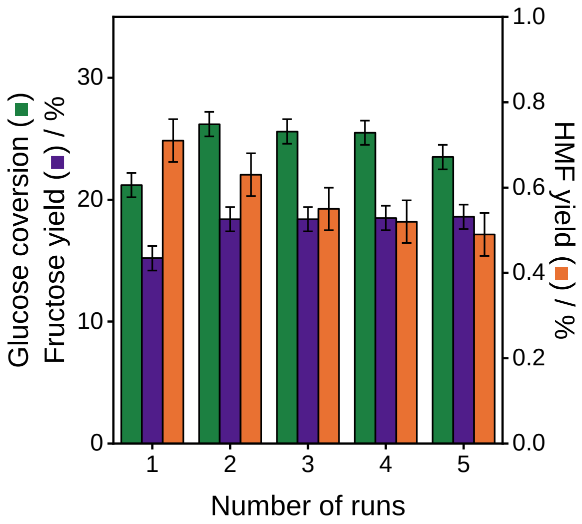

Figure S17. Recycle tests for the glucose conversion in batch using a physical mixture of 15:85 *m*-:*t*-ZrO_2. Reaction conditions: 100 °C; 30:170 mg *m*-:*t*-ZrO_2; 28 mM glucose in 20 mL water, 6 h. Post-reaction catalysts were recovered by centrifugation and regenerated by calcination in a muffle furnace at 700 °C for 2 h (ramp rate of 10 °C min^{-1}).

References:

(1) *https://tsi.com/getmedia/6485a12e-d8bb-4f1a-a74e-3b89738705d6/Identification_of_ZrO2_Phase_w_Raman_Spectroscopy_App_Note_RAMAN-020_A4-web?ext=.pdf*.

(2) Keramidas, V. G.; White, W. B. Raman Scattering Study of the Crystallization and Phase Transformations of ZrO2. *Journal of the American Ceramic Society* **1974**, *57* (1), 22-24. DOI: https://doi.org/10.1111/j.1151-2916.1974.tb11355.x (acccessed 2024/06/12).

(3) Zhao, X.; Vanderbilt, D. Phonons and lattice dielectric properties of zirconia. *Physical Review B* **2002**, *65* (7), 075105. DOI: 10.1103/PhysRevB.65.075105.

(4) Piskorz, W.; Gryboś, J.; Zasada, F.; Cristol, S.; Paul, J.-F.; Adamski, A.; Sojka, Z. Periodic DFT and Atomistic Thermodynamic Modeling of the Surface Hydration Equilibria and Morphology of Monoclinic ZrO2 Nanocrystals. *The Journal of Physical Chemistry C* **2011**, *115* (49), 24274-24286. DOI: 10.1021/jp2086335.

(5) Piskorz, W.; Gryboś, J.; Zasada, F.; Zapała, P.; Cristol, S.; Paul, J.-F.; Sojka, Z. Periodic DFT Study of the Tetragonal ZrO2 Nanocrystals: Equilibrium Morphology Modeling and Atomistic Surface Hydration Thermodynamics. *The Journal of Physical Chemistry C* **2012**, *116* (36), 19307-19320. DOI: 10.1021/jp3050059.

(6) Davis, B. H.; Keogh, R. A.; Alerasool, S.; Zalewski, D. J.; Day, D. E.; Doolin, P. K. Infrared Study of Pyridine Adsorbed on Unpromoted and Promoted Sulfated Zirconia. *Journal of Catalysis* **1999**, *183* (1), 45-52. DOI: https://doi.org/10.1006/jcat.1998.2387.

(7) Saravanan, K.; Park, K. S.; Jeon, S.; Bae, J. W. Aqueous Phase Synthesis of 5-Hydroxymethylfurfural from Glucose over Large Pore Mesoporous Zirconium Phosphates: Effect of Calcination Temperature. *ACS Omega* **2018**, *3* (1), 808-820. DOI: 10.1021/acsomega.7b01357.

(8) Moliner, M.; Román-Leshkov, Y.; Davis, M. E. Tin-containing zeolites are highly active catalysts for the isomerization of glucose in water. *Proceedings of the National Academy of Sciences* **2010**, *107* (14), 6164-6168. DOI: doi:10.1073/pnas.1002358107.

(9) Osatiashtiani, A.; Lee, A. F.; Granollers, M.; Brown, D. R.; Olivi, L.; Morales, G.; Melero, J. A.; Wilson, K. Hydrothermally Stable, Conformal, Sulfated Zirconia Monolayer Catalysts for Glucose Conversion to 5-HMF. *ACS Catalysis* **2015**, *5* (7), 4345-4352. DOI: 10.1021/acscatal.5b00965.

(10) McNeff, C. V.; Nowlan, D. T.; McNeff, L. C.; Yan, B.; Fedie, R. L. Continuous production of 5-hydroxymethylfurfural from simple and complex carbohydrates. *Applied Catalysis A: General* **2010**, *384* (1), 65-69. DOI: https://doi.org/10.1016/j.apcata.2010.06.008.
